# Fully Automated Whole-Head Segmentation with Improved Smoothness and Continuity, with Theory Reviewed

**DOI:** 10.1371/journal.pone.0125477

**Published:** 2015-05-18

**Authors:** Yu Huang, Lucas C. Parra

**Affiliations:** Department of Biomedical Engineering, City College of the City University of New York, New York, NY, USA; University of Iowa, UNITED STATES

## Abstract

Individualized current-flow models are needed for precise targeting of brain structures using transcranial electrical or magnetic stimulation (TES/TMS). The same is true for current-source reconstruction in electroencephalography and magnetoencephalography (EEG/MEG). The first step in generating such models is to obtain an accurate segmentation of individual head anatomy, including not only brain but also cerebrospinal fluid (CSF), skull and soft tissues, with a field of view (FOV) that covers the whole head. Currently available automated segmentation tools only provide results for brain tissues, have a limited FOV, and do not guarantee continuity and smoothness of tissues, which is crucially important for accurate current-flow estimates. Here we present a tool that addresses these needs. It is based on a rigorous Bayesian inference framework that combines image intensity model, anatomical prior (atlas) and morphological constraints using Markov random fields (MRF). The method is evaluated on 20 simulated and 8 real head volumes acquired with magnetic resonance imaging (MRI) at 1 mm3 resolution. We find improved surface smoothness and continuity as compared to the segmentation algorithms currently implemented in Statistical Parametric Mapping (SPM). With this tool, accurate and morphologically correct modeling of the whole-head anatomy for individual subjects may now be feasible on a routine basis. Code and data are fully integrated into SPM software tool and are made publicly available. In addition, a review on the MRI segmentation using atlas and the MRF over the last 20 years is also provided, with the general mathematical framework clearly derived.

## Introduction

The increasing availability of magnetic resonance images (MR images, MRI) at 1 mm^3^ resolution has made it possible to build realistic high-resolution models of individual human heads. Accurate segmentations of the whole-head anatomy are important for the “forward modeling” of current flow in electroencephalography (EEG) and trancranial electric stimulation (TES), as well as their magnetic equivalents—MEG and TMS [1–8]. It is becoming increasingly clear that individual anatomy including complete cerebrospinal fluid (CSF), skull and scalp are key to obtain meaningful source localization in EEG and targeting of TES for individual subjects [1, 5, 9–12].

Unfortunately, currently available segmentation tools are of limited utility for this purpose. Statistical Parametric Mapping version 8 (SPM8, [13]) provides segmentation of the brain, CSF, skull and scalp using the Unified Segmentation algorithm [14], but its field of view (FOV) does not cover the whole head. FieldTrip [15] and the Brain Extraction Tool (BET, [16]) in the FMRIB Software Library (FSL, [17]) can extract the skull and scalp surfaces, but they also only operate on the standard FOV (the brain area only). The FMRIB Automated Segmentation Tool (FAST, [18]) and Integrated Registration and Segmentation Tool (FIRST, [19]) are designed, respectively, only for segmentation of brain tissues and subcortical structures. FreeSurfer [20–23], the Expectation–Maximization Segmentation tool (EMS, [24]), the Atlas Based Classification (ABC) and the EMSegmenter [25–27] in 3D Slicer [28], BrainSuite [29, 30], BrainVISA Morphologist [31, 32], the CIVET segmentation [33], the Sub-Volume Probabilistic Atlases Segmentation tool (SVPASEG, [34]), and the segmentation module in BrainVoyager [35, 36] also only focus on brain tissues. ITK-SNAP [37] and Neuroelectromagnetic Forward Head Modeling Toolbox (NFT, [38]) are semi-automated tools since they need user-specified seed point(s) to start. Commercial software tools, such as ASA (ANT Software B.V., Enschede, Netherlands), Curry (Compumedics NeuroScan, Charlotte, NC), BESA (BESA GmbH, Gräfelfing, Germany) and ScanIP (Simpleware Ltd, Exeter, UK), either are semi-automated or cannot generate (accurate) segmentation for CSF.

Therefore, researchers have attempted several workarounds for the segmentation of the whole head for electromagnetic forward modeling, e.g., combination of different segmentation tools [2, 39], use of computed tomography (CT) images for skull segmentation [40], simple heuristics using thresholding and morphological operations [10, 38], addition of dummy model for the lower head and/or the neck [3], or manual segmentation [1]. In short, these approaches are either too specific, heuristic, or not fully automated for segmenting out CSF, skull, scalp and air cavities. In our view, the simplest way to adapt current automated segmentation tools for this purpose is to extend the atlas representing anatomical prior information to include those non-brain tissues and cover the whole head. This is implemented in SPM8 or in [41]. However, the resulting segmentations still have morphological errors (gray matter, CSF and skull layers are fragmented and some tissue voxels erroneously fall inside other tissues) and the tissue boundaries are unnecessarily rough making subsequent meshing and forward modeling intractable. An automated post-processing routine based on morphological and Boolean operations is proposed in [41], but the technique is *ad hoc* with arbitrary parameters. Since the morphological constraints can be easily encoded by Markov random field (MRF, [42, 43]), here in this work we further improve the method in [41] by combining the atlas with an MRF prior.

The goal of this work is to develop an open-source, fully automated segmentation tool for the purpose of forward modeling. It can separate the whole head into gray matter (GM), white matter (WM), CSF, skull, scalp and air cavities. The method combines the atlas and the MRF prior with a finite mixture (FM) model [44, 45] of image intensities into a single rigorous Bayesian framework. A variational expectation–maximization (EM) algorithm is used with an asynchronous updating scheme that guarantees convergence [46]. We extend the Potts model [47] such that its interaction energy encodes all possible combinations of neighboring tissue types to express strict anatomical constraints. The core algorithm is implemented in the C programing language and integrated into SPM8, where the image registration and bias-field correction are solved using Unified Segmentation [14]. Evaluation is performed on both simulated and real MR images. When comparing to the segmentation tools that are already part of SPM8, we find smoother and less fragmented tissue segmentations without compromising accuracy. As another contribution, this paper summarizes the research on MRI segmentation using atlas and the MRF over the last two decades and presents the general framework with clear mathematical derivations.

## Methods

This section only gives an overview of the segmentation algorithm. For detailed mathematical derivations, the readers are referred to the Appendix, which carefully describes the probabilistic formalism and derives equations that combine spatial and morphological priors.

The probabilistic approach to automated segmentation aims to fit an FM model [45] to the MR image(s) such that the likelihood of observing the image(s) is maximized. This maximum likelihood (ML) estimation is usually implemented by the EM algorithm [48, 49]. When morphological constraints are incorporated as MRF prior to probability model, an extension of ML known as variational EM (VEM) [46, 50, 51] is used. It iterates between estimating the tissue probability for each voxel (E-step) and updating the model parameters (M-step). The iterative update equation for the E-step is given by

$$\begin{array}{c} q\left(x_{i}\right)=\frac{P\left(y_{i}|x_{i},\theta \right)\exp[\frac{1}{2}\beta \sum_{j\in {𝓝}_{i}}\sum_{x_{j}}q\left(x_{j}\right)x_{i}^{T}J_{ij}x_{j}+h_{i}^{T}x_{i}]}{\sum_{x_{i}}P\left(y_{i}|x_{i},\theta \right)\exp[\frac{1}{2}\beta \sum_{j\in {𝓝}_{i}}\sum_{x_{j}}q\left(x_{j}\right)x_{i}^{T}J_{ij}x_{j}+h_{i}^{T}x_{i}]}. \end{array}$$ (1)

Here **x**_*i*_ is a label indicating the tissue class at voxel *i*. For mathematical convenience it is expressed as a *K*-dimensional unit vector with zeros for all *K* tissues, except for the tissue present in that voxel. Eq (1) iterates *q*(**x**_*i*_), which quantifies the estimated probability of finding label **x**_*i*_. After convergence of this iteration, the estimated probability gives the desired segmentation. Variable *y*_*i*_ represents the image intensity in voxel *i* (or several intensities in case of multi-image segmentation) and *P*(*y*_*i*_|**x**_*i*_, ***θ***) is the probability of finding this intensity for a given tissue class. As in many other segmentation algorithms, this probability is assumed to be a Gaussian and its parameters ***θ*** are adjusted to the image during the segmentation process in the M-step (Eq. 3) of the EM algorithm. 𝓝_*i*_ is the set of neighbors of voxel *i*. In this work, we consider only the 6 immediate face-connected neighbors in 3D space. The two terms in the exponential come from the prior model, with matrix **J**_*ij*_ representing the MRF interaction between neigboring voxels and vector **h**_*i*_ representing the effect of the atlas on single voxels. *β* is a weighting constant that adjusts the relative contribution of these two terms and will be tuned using training data (see Evaluation). The quadratic term here can be seen as an extension to the traditional Potts model [47], whose neighboring interaction coefficients **J**_*ij*_ are given by the identity matrix.

Our implementation distinguishes between tissue *class* **x**_*i*_ and tissue *type* ${\bar{\text{x}}}_{i}$ to allow for several classes to belong to the same tissue (e.g. skull bone encompasses cortical and cancellous bone, each with different image intensities). This results in an FM model for each tissue type, which is used in many segmentation algorithms [14, 44, 49]. The complete E-step then follows

$$\begin{array}{c} q\left(x_{i},{\bar{x}}_{i}\right)=\frac{\gamma_{x\bar{x}}P\left(y_{i}|x_{i},\theta \right)\exp[\frac{1}{2}\beta \sum_{j\in {𝓝}_{i}}\sum_{{\bar{x}}_{j}}q\left({\bar{x}}_{j}\right){\bar{x}}_{i}^{T}J_{ij}{\bar{x}}_{j}+h_{i}^{T}{\bar{x}}_{i}]}{\sum_{{\bar{x}}_{i}}P\left(y_{i}|{\bar{x}}_{i},\theta \right)\exp[\frac{1}{2}\beta \sum_{j\in {𝓝}_{i}}\sum_{{\bar{x}}_{j}}q\left({\bar{x}}_{j}\right){\bar{x}}_{i}^{T}J_{ij}{\bar{x}}_{j}+h_{i}^{T}{\bar{x}}_{i}]}, \end{array}$$ (2a)

$$\begin{array}{c} q\left({\bar{x}}_{i}\right)=\sum_{x_{i}}q\left(x_{i},{\bar{x}}_{i}\right). \end{array}$$ (2b)

Here we are interested in the probability of tissue type, i.e., $q({\bar{\text{x}}}_{i})$, since we aim to segment out six tissue types: GM, WM, CSF, skull, scalp and air. The parameters $\gamma_{x\bar{x}}$ are equivalent to the conventional “mixing proportion”. More strictly, they represent the conditional probability of a class belonging to a specific tissue type $P({\text{x}}_{i}|{\bar{\text{x}}}_{i})$ (see Appendix B.8). These parameters will also be adjusted to the image in the M-step. The complete M-step update equations including the update for the mean, covariance and mixing proportion of the Gaussian FM model are given by

$$\begin{array}{c} \mu_{x}=\frac{\sum_{i=1}^{N}q\left(x_{i}=x\right)y_{i}}{\sum_{i=1}^{N}q\left(x_{i}=x\right)}, \end{array}$$ (3a)

$$\begin{array}{c} \Sigma_{x}=\frac{\sum_{i=1}^{N}q\left(x_{i}=x\right)\left(y_{i}-\mu_{x}\right){\left(y_{i}-\mu_{x}\right)}^{T}}{\sum_{i=1}^{N}q\left(x_{i}=x\right)}, \end{array}$$ (3b)

$$\begin{array}{c} \gamma_{x\bar{x}}=\frac{\sum_{i=1}^{N}q\left(x_{i}=x,{\bar{x}}_{i}=\bar{x}\right)}{\sum_{i=1}^{N}q\left({\bar{x}}_{i}=\bar{x}\right)}. \end{array}$$ (3c)

Here *x*_*i*_ = *x* is equivalent to **x**_*i*_ = **x**, representing the tissue class at voxel *i* (see Appendix B.1).

The parameters quantifying morphological and spatial priors, **J**_*ij*_ and **h**_*i*_, respectively, can be obtained from prior data. Suppose we have a dataset of validated segmentations for a group of subjects, and these truth data are spatially normalized, then we can easily get

$$\begin{array}{c} C_{ij}=\left\langle x_{i}x_{j}^{T}\right\rangle , \end{array}$$ (4a)

$$\begin{array}{c} m_{i}=\left\langle x_{i}\right\rangle , \end{array}$$ (4b)

where ⟨…⟩ refers to the sample average over the subjects, and *j* ∈ 𝓝_*i*_. This **m**_*i*_ gives the prior probability of observing a specific tissue type at voxel *i* and is usually referred as “atlas”, or “tissue probability map (TPM)”. **C**_*ij*_ describes the joint probability of co-occurrence of tissues in neighboring voxels *i* and *j*, and will be referred as “tissue correlation map (TCM)” in the sequel. It is not trivial to compute **J**_*ij*_ and **h**_*i*_ from **C**_*ij*_ and **m**_*i*_. In this work we will use the solution for the most simple case of two sites, *N* = 2 (see Appendix C). Up to a scalar that can be captured by parameter *β* it is given by

$$\begin{array}{c} J_{ij}=\log\left[C_{ij}\cdot {\text{diag}}^{-1}\left(m_{j}\right)\right], \end{array}$$ (5a)

$$\begin{array}{c} h_{i}=\log m_{i}. \end{array}$$ (5b)

Note that the mean value **m**_*i*_ is simply the sample estimate of *P*(**x**_*i*_) and the correlation **C**_*ij*_ is the sample estimate of *P*(**x**_*i*_, **x**_*j*_). Consequently, **C**_*ij*_ ⋅ diag^−1^(**m**_*j*_) is the sample estimate of the conditional *P*(**x**_*i*_∣**x**_*j*_) and is column-normalized.

Since the TCM **C**_*ij*_ is dependent on locations, it may not be practical. First, this *local* TCM simply requires too much memory (for a typical 200×200×200-site lattice, each site having a 6×6 matrix with one of its 6 neighbors, that is 200^3^ × 6^3^ × 4bytes ≈ 6.4GB under 32-bit precision). Second, unless the database to compute the local TCM is very large, the values will be poorly estimated. Therefore, we propose a simplified TCM that is independent of locations with **C**_*ij*_ = **C**. Furthermore, since we have prior knowledge from neuroanatomy that some combinations of tissue neigboring are not possible (e.g., WM cannot be neighbor of scalp), we can directly set the corresponding element in **C** to 0. Motivated by the solution for 2 sites, we will also require **C** to be column-normalized and symmetric. Therefore,

$$\begin{array}{c} C=\begin{array}{cc} & \begin{array}{cccccc} \text{GM} & \text{WM} & \text{CSF} & \text{skull} & \text{scalp} & \text{air} \end{array}\\ \begin{array}{c} \text{GM}\\ \text{WM}\\ \text{CSF}\\ \text{skull}\\ \text{scalp}\\ \text{air} \end{array} & \left[\begin{array}{cccccc} d_{1} & C_{1} & C_{2} & 0 & 0 & 0\\ C_{1} & d_{2} & C_{3} & 0 & 0 & 0\\ C_{2} & C_{3} & d_{3} & C_{4} & C_{5} & 0\\ 0 & 0 & C_{4} & d_{4} & C_{6} & C_{7}\\ 0 & 0 & C_{5} & C_{6} & d_{5} & C_{8}\\ 0 & 0 & 0 & C_{7} & C_{8} & d_{6} \end{array}\right] \end{array}. \end{array}$$ (6)

Each row in **C** represents the possible tissue type that voxel *i* may belong to, given that its neighbor *j* is one of the six types indicated by the columns of **C**. Each of the diagonal elements *d*_1_, *d*_2_, …, *d*_6_ is simply 1 minus the sum of other elements in the corresponding column since **C** is column-normalized. Then there are only 8 free parameters *C*_1_, *C*_2_, …, *C*_8_ (each one between 0 and 1) to be learned from the prior data (see Evaluation for details). This **C** will be referred as *global* TCM.

A global **C** is a practical solution to the difficulties of estimating and storing a local TCM, yet it does lose some of the available prior information. For instance, in boundary regions where different tissue types are likely to co-occur, the values of **C** should be quite different from those well inside a tissue (e.g., within scalp, or the air outside of head) where co-occurrence of different tissues is all but impossible. For these interior region the identity matrix, **C** = **I**, used in the conventional Potts model [47], is more appropriate as it favors only neighbors with the same class label. On the boundary, however, co-occurrence of different tissues is likely and thus a more general **C** is needed. To balance between the encoding of this locality information and the potentially huge memory consumption, we propose a simple variant of global TCM, called *regional* TCM, which is simply an identity matrix **I** inside a specific tissue, and the same as global TCM elsewhere. To define the inside-tissue regions, we use the TPM as a reference. For each tissue type, those voxels that have a probability higher than 0.95 in the TPM are treated as the area inside that tissue. In short,

$$\begin{array}{c} C_{\text{regional}}=\{\begin{array}{cc} I, & \text{voxel}\;i\;\text{with}\;{\left(m_{i}\right)}_{x}>0.95,\\ & \forall x\in \{1,2,\cdots ,K\}\\ C_{\text{global}}, & \text{other}\;\text{voxels.} \end{array} \end{array}$$ (7)

Note that for global and regional TCM, Eq 5a simply changes to **J** = log**C**.

## Implementation

The proposed segmentation algorithm is implemented based on the Unified Segmentation algorithm of SPM8 [13]. SPM8 is a software package for Matlab (R2010b, MathWorks, Natick, MA) that is freely available and widely used to analyze brain signals. It implements an extended version of the Unified Segmentation (eUS) algorithm [14], which performs bias-field correction and registration with the atlas as part of the segmentation process. The actual Matlab function for this—“spm_preproc8.m”—is referred to as “New Segment” in the SPM8 manual. It is an extended implementation of Unified Segmentation allowing the use of multimodal data, and a different treatment of the mixing proportions *γ* that is in agreement with the present paper. We leverage this capability by initializing the segmentation algorithm with eUS. Therefore, the value of *y*_*i*_ is the intensity after the optimal bias-field correction, and the voxel locations *i* of the prior parameters **h**_*i*_ and **J**_*ij*_ have been optimally registered to the MRI that is to be segmented, and the initial values of posteriors $q({\text{x}}_{i},{\bar{\text{x}}}_{i})$ and the Gaussian FM model parameters ***μ***_*x*_, **Σ**_*x*_, $\gamma_{x\bar{x}}$ are all initialized by the result of eUS. Note the local TCM is registered to the MRI using the same algorithm applied by the eUS for TPM registration. This step is used in general but was omitted in the evaluation of the simulated data from BrainWeb (see Evaluation), which is already aligned to the TPM/TCM. The implementation is published as a freely available add-on to SPM8, named Morphologically and Anatomically accuRate Segmentation (MARS, available to download at http://neuralengr.com/mars/, the TPM and TCM used for evaluation in this work are also available there). The user can configure all the options (image data, TPM, TCM, running mode, etc.) in a similar batch editor as that in the eUS. The evaluation of this algorithm is based on six-class segmentation. However, the implementation allows any number of classes provided by the corresponding TPM and TCM. In fact, the user can specify any custom TPM/TCM in MARS. With different running modes, the user can select running the original eUS, the eUS with a subsequent MRF-based clean-up (disabled by default in the latest update of SPM8, but can be enabled to a specific level by the user), or eUS followed by the present algorithm.

The E-step (Eq 2a) is implemented in the C programing language for speed and is integrated with Matlab. Each voxel is accessed and updated sequentially in a checkerboard manner. As shown by [46], this sequential (or asynchronous) updating scheme guarantees both theoretical and practical convergence, i.e., the free energy of the system will monotonically decrease to a finite value, which is not guaranteed using parallel (or synchronous) updating. A similar convergence criterion is employed as the one in [46], i.e., the maximal relative change of tissue volumes between consecutive iterations

$$\begin{array}{c} \varepsilon =\max_{x}\frac{\left|L_{x}^{\left(t\right)}-L_{x}^{\left(t-1\right)}\right|}{L_{x}^{\left(t-1\right)}}, \end{array}$$ (8)

where *t* is the iteration number, and *L*_*x*_ is the volume of the *x*th tissue class, defined by $L_{x}=\sum_{i=1}^{N}q(x_{i}=x)$. Convergence is declared once *ɛ* falls below 0.01%. The M-step (Eqs 3a, 3b and 3c) has a same form as that in [14]. Therefore, the same Matlab code is used as the implementation for M-step (mixture coefficient Eq 3c is different from that in [14], but “spm_preproc8.m” actually implements Eq 3c in this paper).

The total time needed to run the algorithm on a typical T1-weighted MRI of size 256×256×181 and 1 mm^3^ resolution is about 60–75 min, in which 10–15 min is consumed by the initialization with eUS. The peak memory load is approximately 50 GB when using local TCM and 2 GB for the global TCM. Note all processing time and memory usages in this work were measured on a machine with a 6-core Intel Xeon E5645 CPU at 2.4 GHz, and 96 GB physical memory.

## Evaluation

### Datasets

Dataset I consists of 20-subject simulated MRIs from BrainWeb (http://brainweb.bic.mni.mcgill.ca/brainweb/, [52]). They are T1-weighted images with 256×256×181 matrix and isotropic 1 mm^3^ voxel size, and simulated for normal brains under a spoiled FLASH sequence with TR = 22 ms, TE = 9.2 ms and 30° flip angle.

Dataset II includes previously published MRI data from three adult male individuals [5], obtained with permission of the authors (Marom Bikson: bikson@ccny.cuny.edu). Head 1 and 2 were scanned on a 3T Siemens Trio scanner (Erlangen, Germany). The T1-weighted images were collected using a gradient echo (GRE) sequence with TE = 4.2 ms, TR = 2250 ms, 256×256×176 matrix for Head 1, and with TE = 2.3ms, TR = 1900 ms, 280×320×208 matrix for Head 2. Head 3 was scanned on a 3T General Electric Signa Excite HD scanner (Fairfield, CT). The T1-weighted images were acquired using a GRE sequence with TE = 2.2 ms, TR = 7.3 ms, 256×256×252 matrix. All images have an isotropic resolution of 1 mm^3^. Dataset III consists of five averaged adult heads from the Neurodevelopmental MRI Database, see [53, 54] for detailed parameters on image acquisition. It was obtained online at http://jerlab.psych.sc.edu/NeurodevelopmentalMRIDatabase/ with permission of John Richards. All human MRIs were obtained as de-identified data and had been collected for previous studies [5, 53, 54].

### Learning of prior model

#### Dataset I

BrainWeb also provides for each of the 20 subjects with the hard segmentation, i.e., each voxel is assigned exclusively to one tissue class. The detailed 12-class hard segmentation was reduced to 6 classes by combining similar tissues together (e.g., fat and muscle integrated with skin). These segmentation ground truth are registered into the same dimension as the MRI scans. Small obvious errors in these hard segmentations were corrected manually in the commercial software ScanIP (version 4.2). The algorithm was evaluated on this dataset using the leave-one-out scheme. At the evaluation for each subject, the hard segmentations from other 19 subjects were used as the prior data to generate the local TCM and TPM using Eqs 4a and 4b, respectively, and then the energy terms were calculated by Eqs 5a and 5b. The MRF weighting constant *β* was tuned using the first subject by running the algorithm for different *β* and selecting the value with the smallest difference (least-square sense) from the known hard segmentation. The optimal value, *β* = 0.1, was then used for all the evaluations using the local TCM. As to the global TCM, it was learned also using the first subject. A general purpose optimization algorithm (Pattern Search [55], available in Matlab) was used to find the **C** in Eq 6 that gives the segmentation results closest to the true hard segmentations in a least-square sense. Reasonable **C** was obtained after 1000 computations of the objective function (the mean square error between generated segmentations and true segmentations), which took approximately 6 days when utilizing the parallel computing ability of Matlab. The resulting **C** was then used for the evaluation of the algorithm on the other 19 subjects.

#### Dataset II and III

The images in Dataset II were first automatically segmented by the eUS, binarized and then manually improved in ScanIP and used as ground truth. As to Dataset III, it provides the truth data with 10-class labeling, which was reduced to 6 classes by combining similar tissues. Since the number of subjects is limited, the local TCM and TPM cannot be generated from the true segmentation, we only evaluated the algorithm on the global TCM and used the TPM from our previous work (see [41], the TPM developed by Dr. C. Rorden). The global TCM **C** was learned using Head 1 in Dataset II by the same procedure as adopted in Dataset I, and then used for the evaluation on the other 7 real MRI volumes. Table 1 shows the learned parameters in the global TCM matrix as in Eq 6.

**Table 1:** The optimal values for the parameters in Eq 6, learned from prior data.

	*C*_1_	*C*_2_	*C*_3_	*C*_4_	*C*_5_	*C*_6_	*C*_7_	*C*_8_
Simulated dataset	0.31	0.27	0.21	0.16	0.02	0.26	0.17	0.24
Real data	0.40	0.20	0.21	0.10	0.001	0.29	0.05	0.30

For the three datasets, the regional TCM was obtained following Eq 7.

### Evaluation metrics

Once the prior model is learned, the algorithm iterates between E-step (Eq. 2) and M-step (Eq. 3) until convergence. The continuous-valued posterior probabilities at convergence are the resulting segmentation and its accuracy was assessed by the fuzzy Dice coefficient [46, 56]. For the *x*th class, it is given by

$$\begin{array}{c} fD_{x}=\frac{2\sum_{i=1}^{N}\sqrt{p\left(x_{i}=x\right)q\left(x_{i}=x\right)}}{\sum_{i=1}^{N}\left[p\left(x_{i}=x\right)+q\left(x_{i}=x\right)\right]}, \end{array}$$ (9)

where *q*(*x*_*i*_ = *x*) is the posterior probability for each tissue on each voxel computed from the algorithm, and *p*(*x*_*i*_ = *x*) is the ground truth. For Dataset I, the soft tissue segmentation for each head was downloaded from BrainWeb and used as ground truth to calculate the fuzzy Dice. The detailed 12-class soft segmentation was reduced to 6 classes by combining similar tissue together, and these ground truth are registered into the same dimension as the MR images. For Datasets II and III, since the non-binary ground truth is not available, the fuzzy Dice was computed using the binary truth. The fuzzy Dice coefficient measures the overlap between the two probability maps, and a value close to 1 indicates more overlap and thus higher performance of the algorithm, while a value close to 0 denotes bad performance.

In most, if not all segmentation problems in medical imaging, there is a trade-off between accuracy and smoothness of the segmented surfaces [57]. In our case smoothness is important in order to generate efficient 3D mesh model of the anatomy after segmentation [41]. Therefore the smoothness of the segmentation results was also evaluated. Gaussian curvature, denote as *K*_*G*_, was used to describe the smoothness of each generated tissue segmentation from the algorithm. Since *K*_*G*_ can be either positive or negative depending on the convexity of the surface of the segmentation, an appropriate measure of smoothness can be obtained by taking square of *K*_*G*_ at each point and integrating over the entire surface, i.e.,

$$\begin{array}{c} sK_{G}=\int \int_{S}K_{G}^{2}ds, \end{array}$$ (10)

where *S* is the tissue surface, approximated by the edge of the segmentation extracted by the Matlab edge detection function. This integral is implemented as a sum of the squared Gaussian curvature across all the voxels on the extracted edge. Gaussian curvature *K*_*G*_ can be obtained from the partial derivatives of the surface *S* [58], which are approximated by the central differences method [59]. *sK*_*G*_ was computed for each generated tissue segmentation, with a smaller value indicating a more smooth segmentation result.

Another useful figure of merit is the porosity of laminar structures such as CSF and skull which are expected to be continuous without any “holes” [41]. To quantify how many holes are there on the surface CSF generated from the segmentation algorithm, we performed a morphological close operation on the CSF and compared how many voxels are changed before and after this operation. More formally, the porosity of the surface CSF was evaluated by

$$\begin{array}{c} \phi =\frac{\left|(I•B)⊻I\right|}{\left|I\right|}, \end{array}$$ (11)

where *I* is the segmented CSF image from the algorithm, *B* is the structure element, • denotes the close operation and ⊻ means exclusive or. Since *I* is non-binary, *B* is constructed as a non-flat cubic structure element, with a Gaussian height with mean of 0 and standard deviation of 1, and the ⊻ operation is conducted in a more general sense, i.e., it returns 0 only when the intensities on a specific voxel are exactly the same before and after the close operation. *B* is set as 11 × 11 × 11 mm^3^ based on visually checking the size of the holes. Therefore, Eq 11 measures the porosity of the surface CSF in terms of the ratio of the amount of changed voxels due to the close operation compared to the total number of voxels in the CSF image. This porosity will tend to be higher if there are many holes on the CSF surface and lower if the CSF is continuous. Eq 11 was also used to measure the porosity of the skull.

## Results

**Fig 1:**
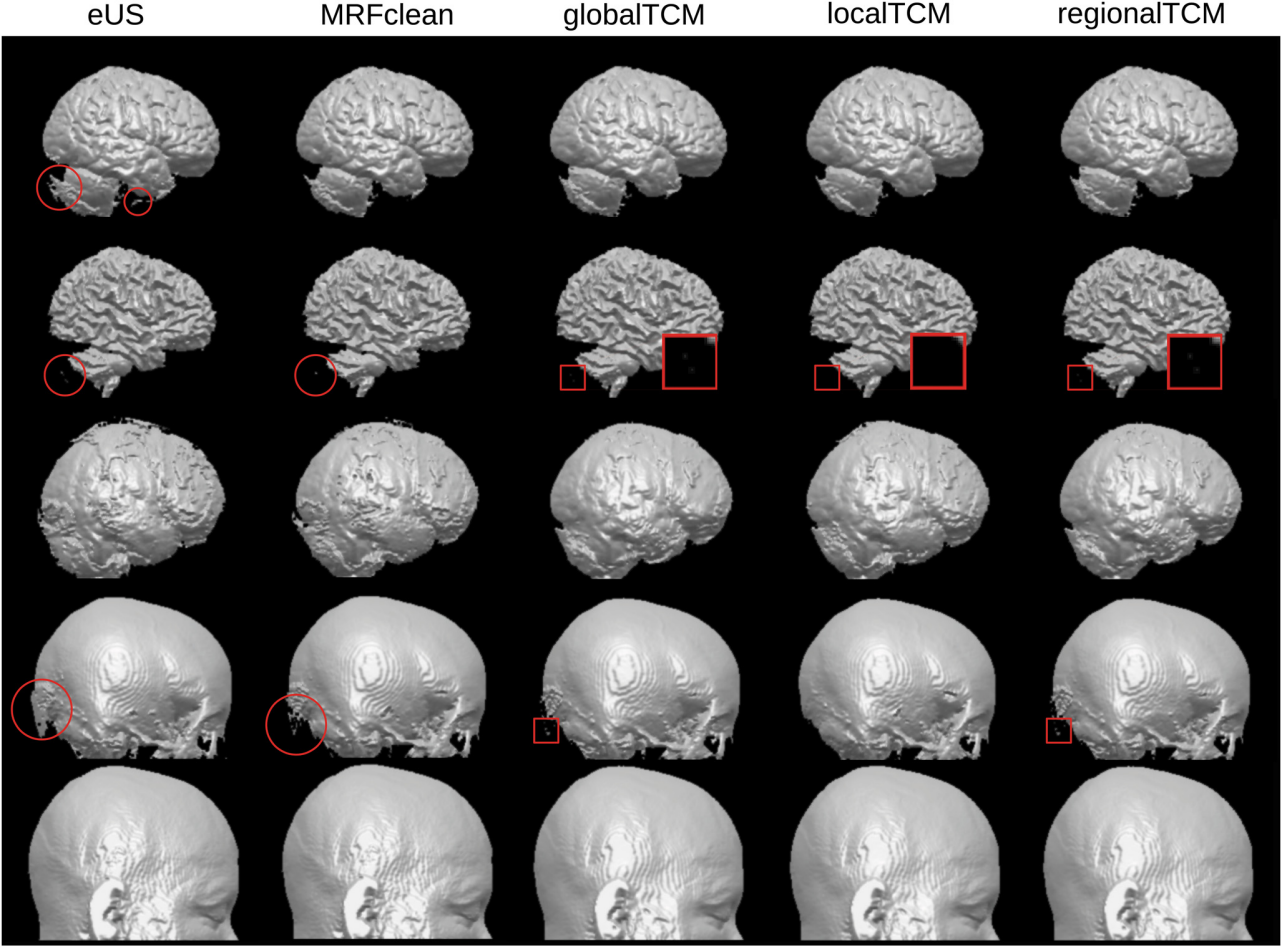
The 3D renderings of the results using different methods on Subject 3 in Dataset I. From the first row to the last row: GM, WM, CSF, skull and scalp. The first column is the results by eUS, the second column is after the MRF-based clean-up, and the third to the last columns are from the proposed algorithm using different TCMs (global, local, regional). Red circles/squares highlight the areas where improvements can be observed.

**Fig 2:**
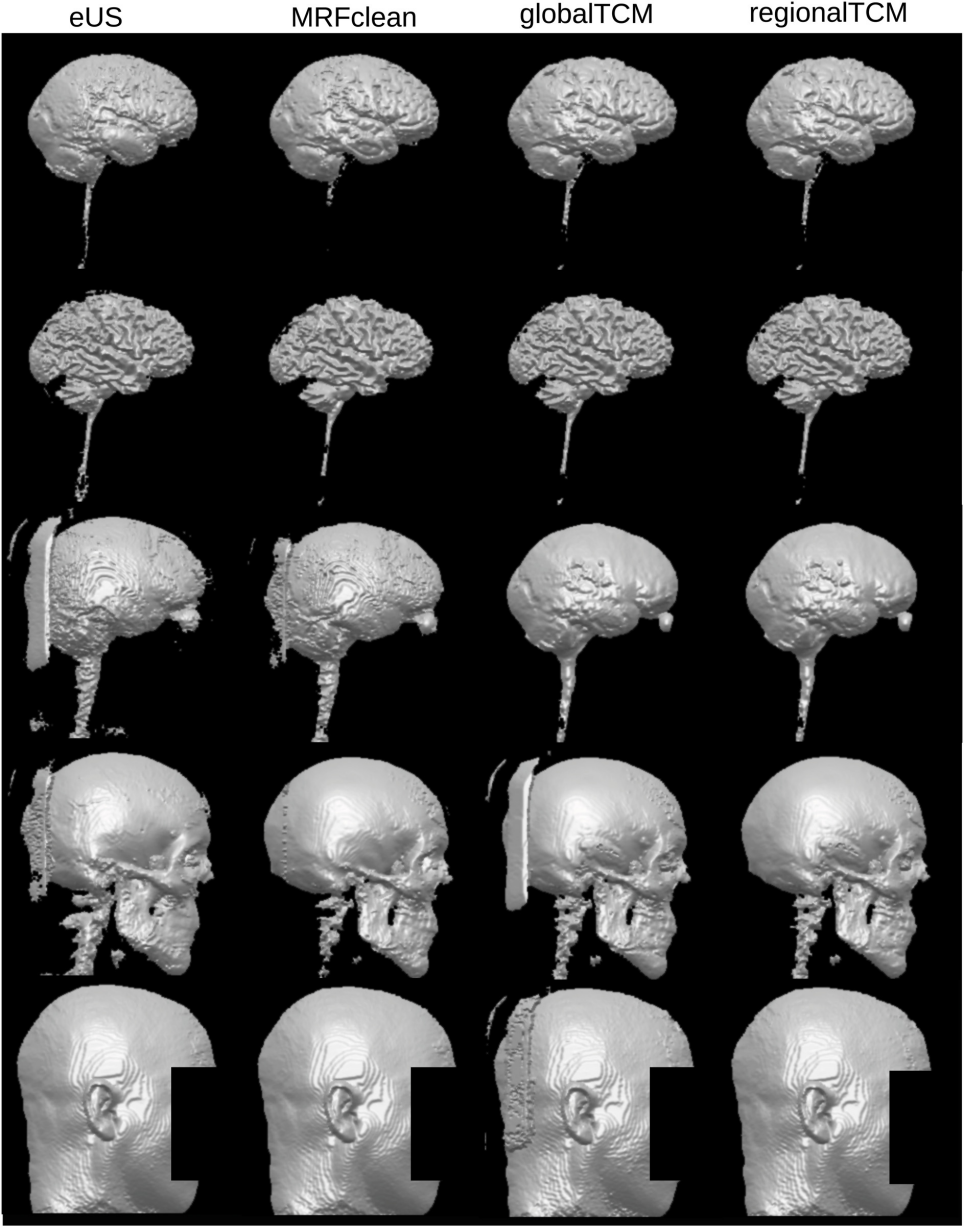
The 3D renderings of the results using different methods on Head 3 in Dataset II. From the first row to the last row: GM, WM, CSF, skull and scalp. The first column is the results by eUS, the second column is after the MRF-based clean-up, and the third and fourth column corresponds to the proposed algorithm using different TCMs (global, regional).

**Fig 3:**
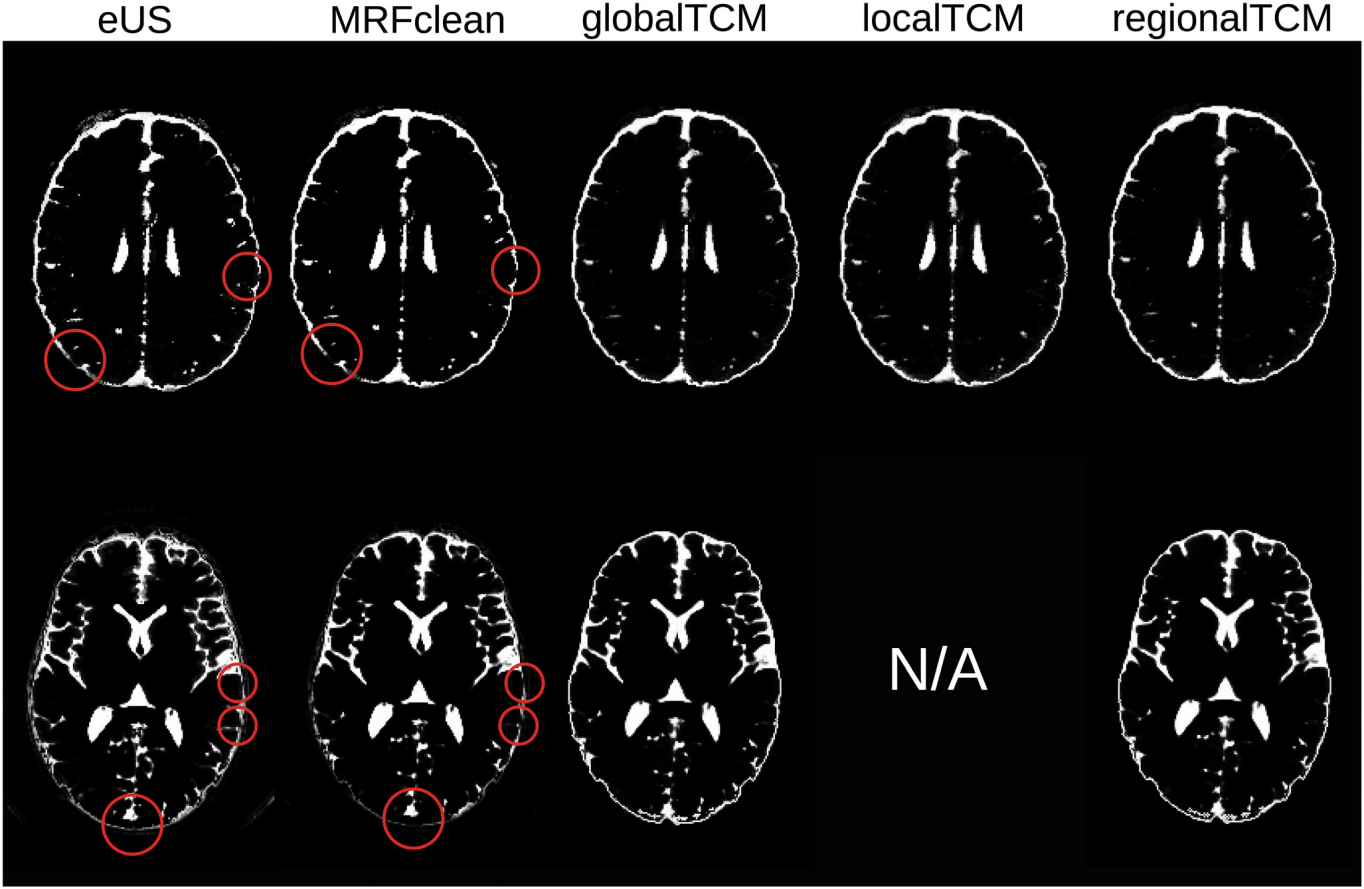
Axial slices of the CSF generated by different methods. The first and second row corresponds to Subject 3 in Dataset I and Head 3 in Dataset II, respectively. Red circles indicate the discontinuities from eUS, which cannot be removed by applying the MRF-based clean-up but can be fixed by the TCM (global, local, or regional).

The proposed algorithm was evaluated on both simulated and real MRI data. For each subject in the simulated data (Dataset I), we compared the performance of the proposed algorithm (with three types of TCM: global, local, regional) to the performance of the eUS in SPM8, and the eUS with an MRF-based clean-up provided by SPM8. For the real data (Datasets II and III), same evaluations were conducted except for the local TCM due to limited prior data. As two examples, Figs 1 and 2 exhibit the 3D renderings of the segmentation results from different methods and for different tissue types. Fig 1 is the results of Subject 3 in Dataset I, and Fig 2 is of Head 3 in Dataset II. The tissue segmentations appear similar across different methods in Fig 1. However, the proposed algorithm improves on smoothness (as indicated by those red circles), especially for CSF and skull. The local TCM is able to remove isolated voxels (indicated by the red squares) due to its detailed location-specific neighborhood constraints. The difference among methods are more obvious in the real MRI (Fig 2), where the proposed algorithm significantly improves smoothness. This head is a good example as the original MRI contains some artifacts in the air outside of the head, leading to large clusters of disconnected voxels in the CSF and skull when using eUS. This segmentation error is removed by the global TCM in CSF, because CSF and air cannot be co-located, as dictated by the 0 probability of CSF–air in the global TCM (Eq 6). However, the probabilities of skull–air and scalp–air are not zero in Eq 6, leading to enhancement of this error in the skull and scalp. In fact, the skull can be adjacent with the inner air (air cavities) but not the air outside of the head, and the scalp only touches outside air at the scalp surface. Fortunately, the regional TCM encodes this local property of neighborhood information and removes these errors. Another important improvement is the continuity of CSF, which is crucial in applications involving current flow [41]. Fig 3 shows that the proposed algorithm fixes the discontinuity problem in the CSF generated by eUS, which cannot be corrected by the MRF clean-up. As a matter of fact, the global TCM (Eq 6) guarantees that the CSF is continuous after segmentation, as indicated by the 0 probability of GM–skull.

**Fig 4:**
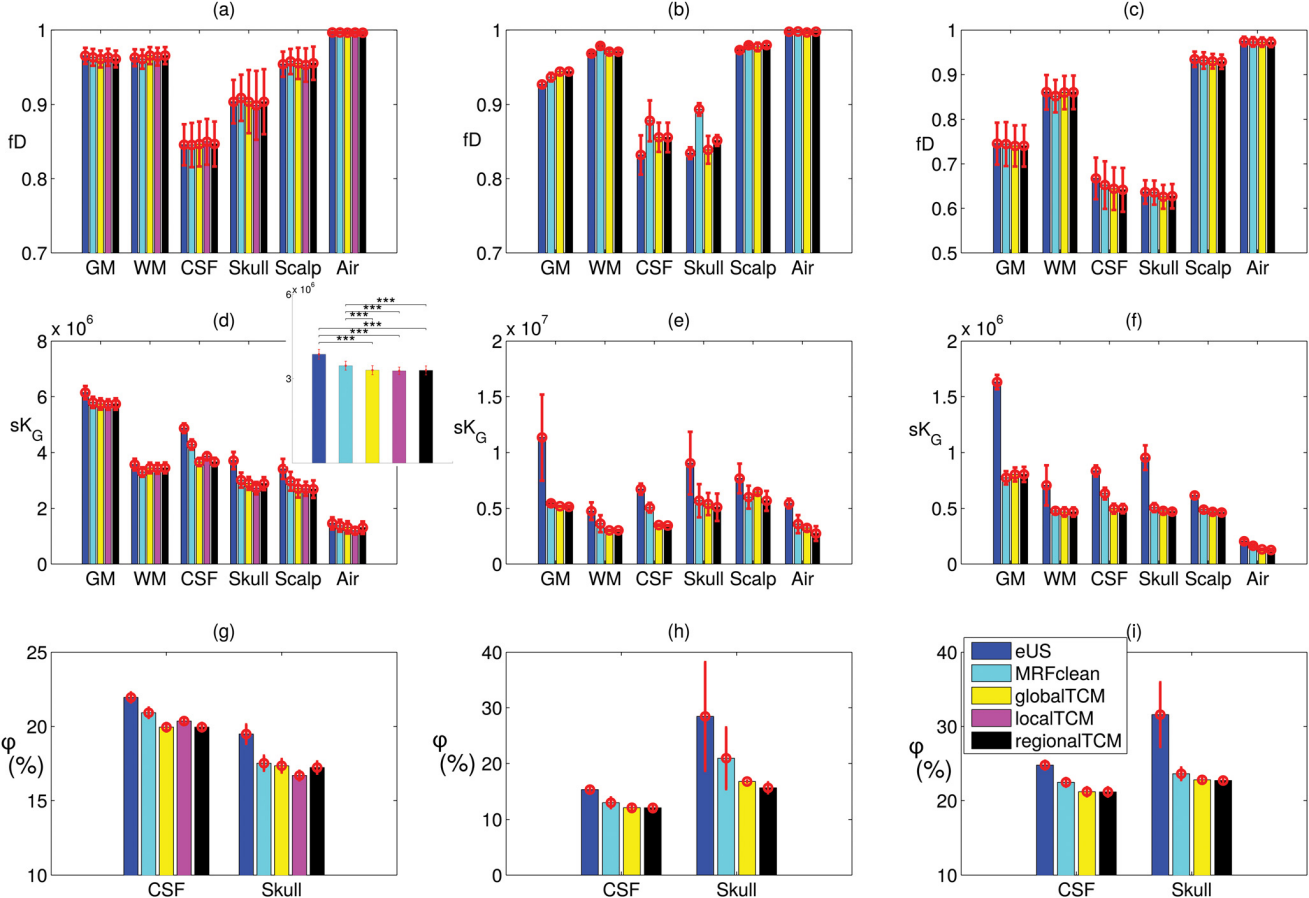
The accuracy, smoothness and porosity of the segmentation results averaged across subjects for Dataset I (Subject 2–20, left column), Dataset II (Head 2 and 3, middle column), and Dataset III (Head 1–5, right column). The red error bars indicate standard deviations across subjects. The inset in (d) shows tissue-averaged quantities. All the colors refer to the legend in (i). (***: significant with *p* < 0.001)

The quantitative assessment of the results is shown in Fig 4. The fuzzy Dice coefficient *fD*, the total squared Gaussian curvature *sK*_*G*_, and the porosity *ϕ* averaged across subjects for different tissues and methods are plotted in the first, second and third row, respectively. The three columns correspond to Dataset I, II and III, respectively. The red error bars indicate the standard deviations of the results across different subjects. Note that since the first subject in Datasets I and II was used for parameter learning (see Evaluation), it is thus excluded in the first two columns in Fig 4. The accuracy of each method seems almost the same in the figure (panels (a), (b), (c)), while the smoothness of the segmentation appears improved by the proposed algorithm (panels (d), (e), (f)). A two-way (tissue, method) repeated measures analysis of variance (ANOVA) was conducted on the accuracy and smoothness of Dataset I. Results show that different methods do not significantly change the accuracy (*F*(4, 72) = 0.55, *p* = 0.70) but do have significant effect on the smoothness (*F*(4, 72) = 1484.83, *p* = 0). Furthermore, a pairwise t-test was conducted between different methods on the tissue-averaged values of *sK*_*G*_. Results show that global, local and regional TCM all significantly improve the smoothness of the segmentation results (*p* < 0.001, see inset of panel (d)). It is obvious that the proposed algorithm gives lower porosity for CSF and skull as compared to the methods in SPM8 (panels (g), (h), (i)). For the porosity results in Dataset I, a one-way (method) repeated measures ANOVA shows that there is significant difference (CSF: *F*(4, 72) = 494.57, *p* = 0, skull: *F*(4, 72) = 380.93, *p* = 0). The pairwise t-test indicates that our algorithm (global, local and regional) significantly reduces the porosity of the CSF and the skull (*p* < 0.001), compared to those obtained from SPM8 (eUS and eUS with MRF clean-up). See panel (g). For Datasets II and III, no statistical testing was conducted due to limited number of subjects.

**Fig 5:**
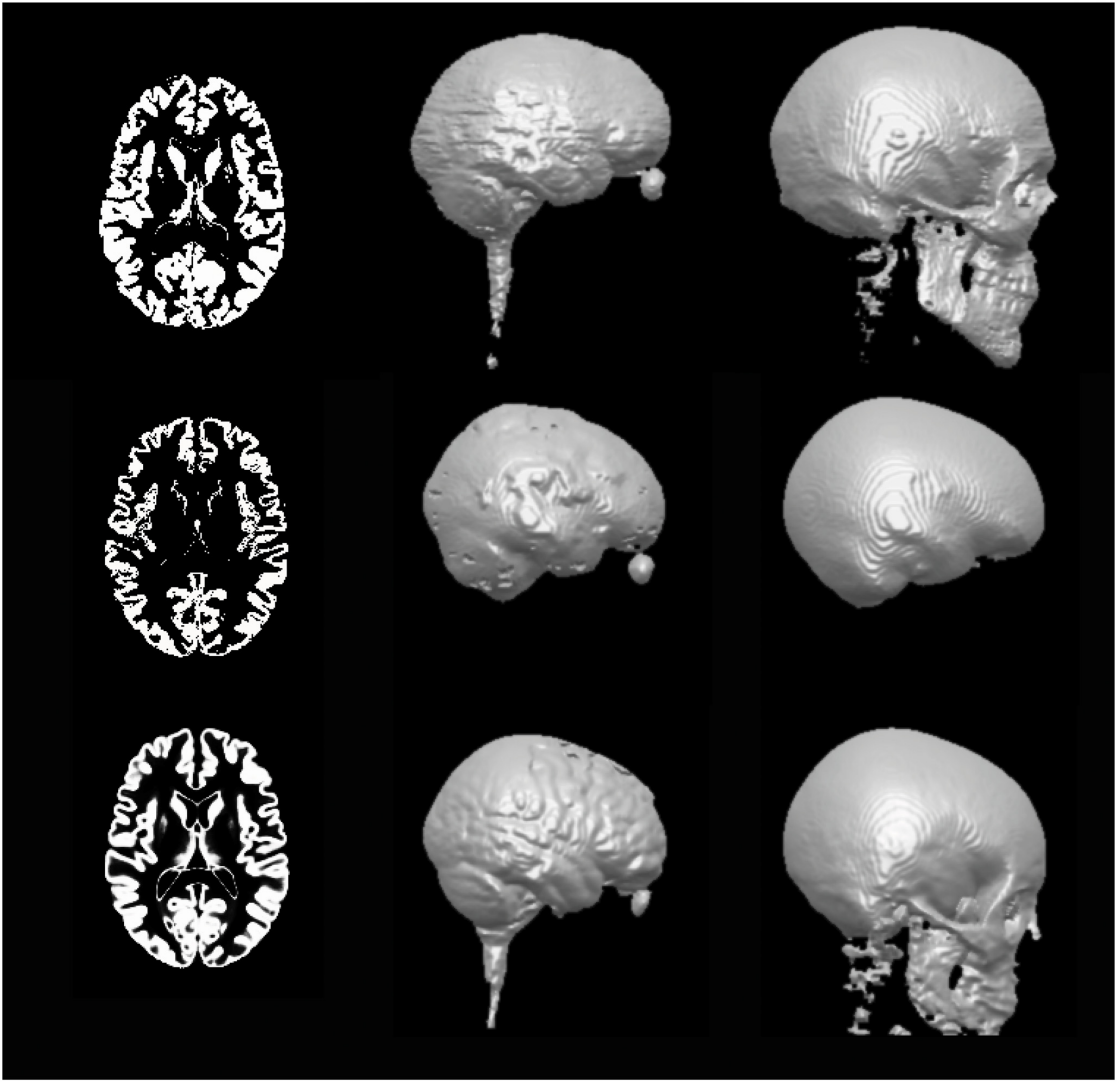
The truth data of Head 3 in Dataset II (first row) and Head 1 in Dataset III (second row). As a comparison, the third row shows the results from the proposed algorithm using global TCM on Head 1 in Dataset III. The columns correspond to axial slices of GM, 3D rederings of the CSF and skull, respectively.

We also note that the accuracy for Dataset III is generally lower than that of Dataset I or II. It is mainly because the quality of the truth data provided by Dataset III is lower compared to the ground truth we manually improved in Datasets I and II. For example, Fig 5 shows the truth images of Head 3 in Dataset II and Head 1 in Dataset III in the first two rows, respectively. It is obvious that the latter has lower quality, as shown in the noisy GM slice, discontinuous CSF surface, and incomplete skull volume. Qualitatively speaking, the results from the proposed algorithm using global TCM on this head are more accurate and realistic than its truth data, as shown in the third row of Fig 5. However, we admit that although the ground truth in Dataset II is obtained by manual improvement, it is by no means a gold standard. In fact, it has the same origin as the automated results, i.e., initially generated by the eUS in SPM8. Therefore, it is in some sense biased. Nevertheless, the main argument here is that based on the same truth, the accuracy is not significantly altered by different methods in Dataset I, and meanwhile the proposed algorithm can achieve better smoothness and lower porosity of the segmentation.

## Discussion

Automated MRI segmentation combining a probabilistic model for image intensity with an atlas and MRF regularization was first proposed in the late 1990s [24, 26], with implementations in many neuroimaging software tools, such as EMS [24], ABC and EMSegmenter [25–27] in 3D Slicer, FAST [18], Freesurfer volumetric approach [22, 23], the SVPASEG [34], and Atropos [60]. Unfortunately these tools only focus on brain tissues. Moreover, Freesurfer volumetric approach only present a heuristic derivation for its update equations, and does not include any EM iteration (the Gaussian parameters are obtained from prior data and never updated). This limits its use to imaging modalities (and scanning parameters) for which calibration data is available. In contrast, the EM algorithm used here estimates the intensity parameters and can thus be applied to arbitrary image modalities (T1, T2, FLAIR, CT, etc.) without concern for historical data or even calibration and image intensity artifacts. The algorithm implemented in the Atropos software is conceptually similar to the present work. It is an open-source segmentation tool that combines user-defined atlas, MRF prior and image model into the EM iterations. In the present work we have focused in addition on smoothness and porosity of the resulting segmentations, which is crucially important for current-flow modeling or lead-field calculations.

This paper summarizes the framework of probabilistic automated MRI segmentation developed over the last two decades and presents it with a systematic mathematical derivation (see Appendix). When applied to the FM model with an atlas as prior one obtains the update equations used in SPM8 (“New Segment” function). When including in addition an MRF as part of the EM update we obtain the algorithm of [24]. To our knowledge the present paper provides the first systematic and complete derivations of these update equations.

The present paper proposes an extension to the conventional energy function of the MRF, namely, an extended Potts model that is consistent with the maximum entropy prior that specifies mean and covariance on historical data. The method is implemented using an updating scheme that guarantees convergence, and is integrated into SPM8 where the bias field correction and image registration are handled by the “New Segment” function. Both simulated and real MRI data are used to evaluate the algorithm. The proposed method is shown to successfully improve the smoothness and porosity without compromising accuracy. More importantly, since the implementation can segment out 6 tissues including CSF, skull, scalp and air in an FOV covering the whole head, it can be used for forward modeling for TES/TMS/EEG/MEG.

Smooth segmentations and a CSF without any discontinuity are crucial for current-flow modeling, in particular modeling of TES [41]. Previously, we achieved this by using morphological and Boolean operations as a post-processing step to the segmentation results [41]. Such heuristic algorithms have a number of parameters that have to be selected judiciously. In this work, we prevent *ad hoc* procedures with arbitrary parameters by incorporating spatial priors in the TCM (see Eq 6), which regularizes the segmentation at each iteration. Importantly, by combining morphological priors into the segmentation algorithm, spatial considerations can be taken into account while at the same time respecting the intensity information. Post-processing operations in contrast typically smooth the segmentation results while entirely ignoring the original image(s). The proposed algorithm can generate tissue segmentations with significantly lower porosity compared to those from SPM8. Therefore, the results from this algorithm can be used for TES modeling directly without any need of automated clean-up or manual correction.

We would like to point out some differences in the use of the mixing proportion variable *γ* in this work and that of [14]. By enforcing normalization of the mixing proportions $\gamma_{x\bar{x}}$ for each tissue type $\bar{x}$, we are biasing the segmentation to match the relative volume fractions of the TPM. This bias may introduce errors. In stroke or geriatric populations, for instance, subjects present typically with substantially larger CSF fraction. Enforcing tissue volume fraction of the normal TPM may not be desired in these populations. In such a case, the approach as in Eq. 27 in [14] may be preferable to our Eq 3c. [14] introduces an additional free parameter (also denoted as *γ* in [14], but with different meaning) to globally rescale and re-normalize the atlas, thus accounting for variation of tissue volumes fractions in different individuals. Interestingly, SPM8 implements Eq 3c and only SPM12 implements as originally proposed in [14]. In order to stay within a strict probabilistic model yet account for different volume fractions, we propose instead to construct an atlas that matches the target population.

Except for SPM8, we did not compare our algorithm with the other software tools mentioned above, simply because they cannot generate segmentations for non-brain tissues in an FOV covering the whole head. For this comparison, we would need to extend the FOV and tissue types for all the candidate tools, which is beyond the scope of this work. Similarly, except BrainWeb, we did not use other public datasets that are commonly adopted for evaluations in the neuroimaging community (e.g., IBSR, LPBA40 [61], segmentation validation engine [62]), because these datasets do not provide ground truth for non-brain tissues. We did however provide results for 8 subjects with non-brain ground truth (3 from our lab and 5 from the Neurodevelopmental MRI Database of John Richards).

Since the TPM provides the anatomical information, and the TCM gives the neighborhood constraints, the accuracy of this algorithm is strongly dependent on the quality of the two maps. For reliable estimates, the size of the dataset used to generate these maps may be important, in particular for the TCM. In this work, only 19 subjects are available, which is small compared to the 36 × 6 parameters in **C**_*ij*_ that are to be estimated for each location (6 stands for 6 face-connected neighbors). Additionally, the exact calculation of the MRF parameters **J**_*ij*_, **h**_*i*_ from the observed correlations **C**_*ij*_ and means **m**_*i*_ is an open problem. The analytic solution for the trivial 2-site lattice which we used here likely overestimates the strength of interaction (as it ignores the indirect influence of distant neighbors and loops) and thus we had to adjust it by tuning parameter *β*. The correct solutions should be obtained from solving Eq 6, which is known as the “inverse Ising problem” in statistical physics. Exact solutions are known for dependencies with an acyclic graph structure (e.g., tree structure, [63]), and approximate analytical solution for network with loops (e.g., regular lattice, [64]). However, these solutions are only available for 2-state variables of the Ising model, where **J**_*ij*_ and **h**_*i*_ are only scalars. In this work, we are dealing with a *K*-state variable **x**_*i*_, for which theoretical solutions are not yet available. Thus we have taken a pragmatic approach and have made the parameter **J** independent of locations *ij* and tuned it so that we obtain the best possible segmentations on known data. Finally note that the VEM algorithm we used here to compute approximate posterior probabilities may find improvements with newer techniques for computing probabilities in networks with loops such as “loopy belief propagation” and “tree-reweighted message passing” [65].

## Appendix

The probabilistic formalism for image segmentation has a long history starting in the 1980s, if not earlier, and encompasses efforts to include spatial priors through an atlas as well as morphological priors using MRF. Parameters of the models are adjusted to the images using the EM algorithms and variations thereof. The earliest work we found on combining morphological and spatial priors in this formalism are [24, 26]. The goal of this appendix is to summarize this body of work with a consistent notation and provide a complete derivation of the EM update equations, which we have not been able to find elsewhere in the literature. This appendix starts with a review of work in this field, introduces notation and formalism, derives the EM update equations, and discusses a few novel choices of our implementation.

## A Review of probabilistic model for image segmentation

Probabilistic inference techniques have been developed for MRI brain segmentation over the years (e.g., [14, 18, 24, 25, 34, 49, 60]). The key idea is to establish a probabilistic model of the image intensity distribution using a finite mixture (FM) model [45]. Automated segmentation is then a process of fitting the FM model to maximize the likelihood of observing the MR image(s). Since the true tissue types of voxels are unknown (“missing data”), this maximum likelihood (ML) estimation is usually implemented by the expectation–maximization (EM) algorithm [48]. Early work dates back to [49] and was reviewed in [44]. In [25] the EM algorithm is also used to correct intensity inhomogeneities and segment the MRI iteratively, which was later implemented as the EMSegmenter. However, such FM models only use intensity and ignore available spatial contextual information, as pointed out by [18]. Two approaches have been independently pursued to incorporate prior knowledges into the ML estimation process.

The first type of prior model is a brain atlas, which quantifies for each voxel location the prior likelihood of observing a given tissue type. It is built by registering many MRI volumes into a common standard space, assigning a specific tissue type to each voxel and then averaging across these volumes [66, 67]. Such an atlas is also known as tissue probability map (TPM) as it quantifies the anatomical prior probability irrespective of the observed intensity in a given image. Since the atlas is in a standard space, image registration is required in order to map the atlas onto the image that is to be segmented. Yet, mapping the image requires an estimate of what tissue is contained in each voxel, i.e. an estimated segmentation. An integrative approach to resolve this interdependence is the Unified Segmentation algorithm [14], which is now publicly available in the Statistical Parametric Mapping 8 (SPM8) software [13]. It combines registration, segmentation as well as bias-field correction into a single optimization problem (bias-field correction compensates for intensity inhomogeneities often observed in MRI). A similar approach is taken in [68] and [27] where the EM algorithm is used to optimize segmentation and registration parameters simultaneously, and registration is achieved in a hierarchical manner such that detailed anatomical structures can be recursively differentiated. An implementation of this method is made into the EMSegmenter in 3D Slicer [28].

The second type of prior knowledge that has been leveraged for segmentation is neighborhood relationships (e.g., not all tissue types can be adjacent to each other). These relationships have been quantified using a Markov random field (MRF), which assigns an “energy” to combinations of neighboring tissue types [42, 43]. Such an energy effectively provides (prior) probabilities making some combinations highly likely (low energy) and others very unlikely (high energy), irrespective of image intensity. MRF has been widely used to regularize the segmentation to ensure spatial consistency [18, 24, 26, 34, 60, 69–72]. Unfortunately, computing posterior probabilities exactly becomes intractable once the spatial dependencies are introduced into the prior probabilities with an MRF. Researchers have proposed a variety of solutions to this problem. Monte Carlo simulation by drawing samples from the MRF [73, 74] is a classical numerical technique to approximate the posterior probability of the voxel label, but it is not computationally efficient. Another classical approach, based on the mean-field theory, is to approximate the influence of the neighbors onto a voxel using their mean influence. The posterior distribution of voxel labels can then be approximated by the product of voxelwise marginals [75]. This has been successfully applied to MRI segmentation [24, 70, 74]. A variant of mean-field approximation is proposed by [18], where a voxel is affected by the most probable label of each of its neighbors (the pseudo-likelihood maximization as in [47]). This has been used in many segmentation tools incorporating the MRF, such as the FMRIB Automated Segmentation Tool (FAST) in FSL [17], the CIVET segmentation [33], and Atropos [60]. Recently, [46] pointed out that both [75] and [18] adopt a synchronous updating scheme which is not guaranteed to converge. If instead voxels are updated sequentially (asynchronous update), convergence of the approximated EM in [75] and [18] is guaranteed. Finally, we note that the interaction energy used in most MRF are based on the Potts model [47] which cannot express constraints for all possible combinations of neighboring tissue types.

These two types of priors have also been combined into a single inference framework and implemented in a number of software tools (see Discussion in the main text). However, there is no publication that summarizes the complete formalism with a consistent notation. This is the goal of the next few sections.

## B Derivation of the variational EM formalism

We start this section by introducing notation, and then we provide an outline of the estimation process. We will review the ML optimization scheme which is applicable when only an atlas is used as prior. To incorporate neighborhood priors we will need an extension of ML known as variational EM (VEM). Then we present the probabilistic model for image intensity and prior probabilities. With these we can then derive the entire estimation algorithm.

### B.1 Notation

Consider an isotropic three-dimensional regular lattice system 𝓘 with *N* sites which are indexed by *i* ∈ {1, …, *N*}. An observed image **Y** is defined on this lattice, where each site represents a voxel of intensity *y*_*i*_. Note for multimodal data (T1, T2, proton density images, etc.), **Y** is a vector field and thus each site contains a vector of voxels **y**_*i*_ = (*y*_*i*1_, *y*_*i*2_, …, *y*_*iM*_)^*T*^, where *M* is the total number of observed images and ^*T*^ is transpose. A labeling (or state) of the lattice system, i.e., the hard segmentation, is denoted by **X**. Assuming there are *K* tissue classes, **X** assigns each site a specific value *x* ∈ (1, …, *K*), which we denote as *x*_*i*_ = *x*. It will also be convenient to express the class label as a *K*-dimensional unit vector **x** with a 1 in the *x*th element and 0 elsewhere, and the assignment of a specific label in location *i* is denoted as **x**_*i*_ = **x**. We use these two equivalent notation styles interchangeably in this paper. A sum over *x* or a sum over **x** represents the same sum over *K* possible values. The set of all possible labelings is 𝓧. The goal of the segmentation is to estimate the underlying hidden labeling **X** that generates the observed image(s) **Y**.

### B.2 Estimation Approach

We will formulate a probabilistic model for the observed image intensity *P*(**Y**∣***θ***), where ***θ*** are parameters describing the brightness distribution for each tissue. Note here ***θ*** is a general placeholder, representing mean and standard deviation of the Gaussian distribution for each tissue class. When the mixture model is introduced for each tissue type (Section B.8), ***θ*** also includes the mixing proportions. In fact, given knowledge of the tissue within each voxel, the intensity distribution should be more narrowly specified as *P*(**Y**∣**X**, ***θ***) (see Appendix B.5.1). Of course we do not know the actual tissue class in each location, but we will specify an *a priori* probability *P*(**X**) of observing a specific segmentation **X** so that the probability of observing the image(s) is

$$\begin{array}{c} P\left(Y|\theta \right)=\sum_{X\in 𝓧}P\left(Y|X,\theta \right)P\left(X\right). \end{array}$$ (12)

This expression also defines the likelihood function of the unknown parameters ***θ***. To estimate these parameters for a given image **Y**, one often uses ML estimation, i.e., the estimated parameters θ* are those that maximize Eq 12. In principle, once these parameters have been fit to the data by ML estimation, one can then find the best segmentation by maximum *a posteriori* (MAP) estimation, i.e., the segmentation X* that maximizes

$$\begin{array}{c} P\left(X|Y,\theta^{*}\right)=\frac{P\left(Y|X,\theta^{*}\right)P\left(X\right)}{\sum_{X\in 𝓧}P\left(Y|X,\theta^{*}\right)P\left(X\right)}, \end{array}$$ (13)

Unfortunately, with *N* = 10^6^ voxels and *K* = 6 tissue classes, it is not feasible in practice to compute this posterior for all possible *K* ^*N*^ values of **X** to select the one with the highest probability. To make the problem tractable, it is customary to make two important simplifications: 1) assume that intensity is not affected by tissues from neighboring voxels; 2) approximate the posterior by a distribution that factorizes over voxels (known as mean-field approximation [75]). With these simplifications the posterior factorizes and the sum in Eq 12 has a manageable *NK* terms. To make this approximation as tight as possible, the parameters are optimized using a formalism known as VEM, which is a generalization of the ML formalism [51]. The resulting posterior represents then the desired segmentation, namely, for every voxel *i* we will have computed the (approximate) probability that this voxel belongs to a particular tissue class *x*, given the observed image intensities: *P*(**x**_*i*_ = **x**∣**Y**, θ*).

Now let us make this overall approach more specific.

### B.3 ML optimization for factorial prior (spatial prior)

In ML the parameters ***θ*** are selected so as to maximize the likelihood of the observed image(s) **Y**, or equivalently, the negative log-likelihood is minimized, i.e.,

$$\begin{array}{ccc} \theta^{*} & = & \underset{\theta }{\text{argmax}}\;\;P(Y|\theta )=\underset{\theta }{\text{argmin}}\;\;-\log P(Y|\theta )\\ & = & \underset{\theta }{\text{argmin}}\;\;-\log\sum_{X\in 𝓧}P\left(Y,X|\theta \right). \end{array}$$ (14)

The sum over the state space 𝓧 is tractable under the following two assumptions: 1) the intensities depend only on the tissue in each voxel,

$$\begin{array}{c} P\left(Y|X,\theta \right)=\prod_{i=1}^{N}P\left(y_{i}|x_{i},\theta \right), \end{array}$$ (15)

and 2) the prior probability of finding a specific tissue in a voxel is independent of what tissue is in other voxels,

$$\begin{array}{c} P\left(X\right)=\prod_{i=1}^{N}P\left(x_{i}\right). \end{array}$$ (16)

We call such a prior an “atlas”, or TPM, since the prior probability of finding a certain tissue depends only on the location of the voxel. Under these two assumptions the MAP estimation becomes trivial. One only needs to compute the posterior probability for each voxel

$$\begin{array}{c} P\left(x_{i}|y_{i},\theta^{*}\right)=\frac{P\left(y_{i}|x_{i},\theta^{*}\right)P\left(x_{i}\right)}{\sum_{x_{i}}P\left(y_{i}|x_{i},\theta^{*}\right)P\left(x_{i}\right)}, \end{array}$$ (17)

where the sum over **x**_*i*_ implies a sum over the *K* possible values **x**_*i*_ = **x**, and then pick the most probable label for each voxel, or simply use this posterior probability as the desired segmentation. So in the case that only an atlas is used as the prior, one can perform exact Bayesian inference. However, if one would like to use morphological priors, in which the probability of observing a certain tissue class depends on the tissues surrounding that voxel, then the prior can no longer be factorized and Eq 13 becomes intractable.

### B.4 Variational EM for Markov random field (neighborhood prior)

To address this problem, the VEM [50, 51] introduces an auxiliary probability distribution *Q*(**X**) and defines an upper bound to the negative log-likelihood in Eq 14, known as the free energy function *F*(*Q*, ***θ***)

$$\begin{array}{ccc} -\log P(Y|\theta ) & \leq & -\log P(Y|\theta )\\ & & +\sum_{X\in 𝓧}Q\left(X\right)\log\frac{Q(X)}{P(X|Y,\theta )}=F\left(Q,\theta \right). \end{array}$$ (18)

The first term in this expression is the negative log-likelihood as before. The second term is the Kullback–Leibler (KL) divergence *D*(*Q*‖*P*_**X**|**Y**, ***θ***_) which is minimal and equals zero for *Q*(**X**) = *P*(**X**|**Y**, ***θ***). For this limiting case, maximizing the likelihood is identical to minimizing the free energy. More generally, minimizing the free energy *F*(*Q*, ***θ***) will (approximately) maximize the likelihood *P*(**Y**∣***θ***) while finding the closest approximation *Q*(**X**) for the posterior probability *P*(**X**|**Y**, ***θ***). In the VEM algorithm one therefore minimizes the free energy, and updates for *Q* and ***θ*** in alternating steps:

$$\begin{array}{c} \text{E-step:}\;\;Q(X)\leftarrow \underset{Q}{\text{argmin}}\;F(Q,\theta ), \end{array}$$ (19a)

$$\begin{array}{c} \text{M-step:}\;\;\theta \leftarrow \underset{\theta }{\text{argmin}}\;F(Q,\theta ). \end{array}$$ (19b)

The key idea in order to simplify the sum over 𝓧 is to constrain the auxiliary probability distribution to have factorial form

$$\begin{array}{c} Q\left(X\right)=\prod_{i=1}^{N}q\left(x_{i}\right). \end{array}$$ (20)

In this scenario, minimizing the KL divergence *D*(*Q*‖*P*_**X**|**Y**,***θ***_) aims to find the closest factorial approximation of the posterior *P*(**X**|**Y**, ***θ***). This approximation is equivalent to the mean-field approximation [46, 51, 75] where the effects of the neighbors are summarized into a mean effect on the isolated voxel. Using this approximate posterior in Bayes rule (Eq 13) is known as approximate Bayesian inference [51]. Detailed equations for this will be given in Appendix B.7 and Appendix B.9 after we parametrize the prior probability and the conditional intensity distribution.

### B.5 Parametrizations

To make further progress we now have to define the prior probability *P*(**X**) and the conditional intensity distribution *P*(*y*_*i*_|**x**_*i*_) and how these depend on the parameters that are to be fit to a given MR image **Y**.

#### B.5.1 Image intensity

We will assume that the image intensity is determined, up to the Gaussian noise, by the tissue class,

$$\begin{array}{c} P\left(y_{i}|x_{i}=x,\theta \right)=\frac{1}{\sqrt{2\pi \sigma_{x}^{2}}}\exp[-\frac{{\left(y_{i}-\mu_{x}\right)}^{2}}{2\sigma_{x}^{2}}], \end{array}$$ (21a)

where of all the parameters ***θ*** only the mean (*μ*_*x*_) and standard deviation (*σ*_*x*_) of the *x*th tissue class are relevant. For multimodal data, the Gaussian probability density function has a vector form as

$$\begin{array}{c} P(y_{i}|x_{i}=x,\theta )=\frac{1}{\sqrt{{\left(2\pi \right)}^{M}|\Sigma_{x}|}}\exp[-\frac{1}{2}{\left(y_{i}-\mu_{x}\right)}^{T}\Sigma_{x}^{-1}\left(y_{i}-\mu_{x}\right)], \end{array}$$ (21b)

where ***μ***_*x*_ and **Σ**_*x*_ are the mean vector and the covariance matrix.

#### B.5.2 The priors

Generally speaking, both the atlas information and the neighborhood relationships can be expressed as prior probability within the formalism of the MRF. First note that, like any other probability, the prior can be defined in terms of a “potential” function *U*(**X**) as [47]

$$\begin{array}{c} P\left(X\right)=\frac{1}{Z}\exp\left(-U\left(X\right)\right), \end{array}$$ (22)

where $Z=\sum_{\text{X}\in 𝓧}\exp(-U(\text{X}))$, known as the partition function, is a constant that ensures the probability is properly normalized. This potential, known as Gibbs potential in the context of statistical mechanics, takes on a particularly simple form if we can assume, as in the MRF, that variables are affected only by a set of neighbors. The Gibbs potential can be written as the sum over individual voxels and all neighbor pairs [43]

$$\begin{array}{c} U\left(X\right)=\sum_{i=1}^{N}V_{i}\left(x_{i}\right)+\frac{1}{2}\sum_{i=1}^{N}\sum_{j\in {𝓝}_{i}}V_{ij}\left(x_{i},x_{j}\right), \end{array}$$ (23)

𝓝_*i*_ denotes the set of neigbors of voxel *i* and the factor of 1/2 compensates for the double-counting of pairs. In this work, we consider only the 6 immediate face-connected neighbors in 3D space. In statistical mechanics [42], *V*_*i*_(**x**_*i*_) is known as the energy of an “external field” acting on individual voxels *i*, and *V*_*ij*_(**x**_*i*_, **x**_*j*_) is the energy of the pairwise interaction between neighboring voxels *i* and *j*. The essence of Eq 23 is that the joint prior probability factorizes over voxels and pairs of voxels.

In the previous section we have seen that the spatial prior can be factorized over voxels (see Eq 16). If only the external field is acting on the voxels (i.e., *V*_*ij*_(**x**_*i*_, **x**_*j*_) = 0), then we can identify the first order potential as *V*_*i*_(**x**_*i*_) = −log*P*(**x**_*i*_). Here *P*(**x**_*i*_) is just the prior probability determined by the atlas (or TPM). In order not to confuse the notation in the sequel, we denote the external field with a new symbol (*h*_*i*_)_*x*_ such that (*h*_*i*_)_*x*_ = log *P*(**x**_*i*_ = **x**), or expressed as a vector for voxel *i*

$$\begin{array}{c} h_{i}=\log P\left(x_{i}\right). \end{array}$$ (24)

Then we can write the first order potential as

$$\begin{array}{c} V_{i}\left(x_{i}\right)=-h_{i}^{T}x_{i}. \end{array}$$ (25)

Eq 25 tells us that the external energy is simply the negative logarithm of the TPM (when only the external field is presented), such that assignments with low probability in the TPM are energetically costly. As to the interaction energy *V*_*ij*_, it is often defined in terms of Kronecker delta, *V*_*ij*_(**x**_*i*_, **x**_*j*_) = −*δ*(**x**_*i*_, **x**_*j*_), i.e., the potential is zero unless both voxels have the same class label. Thus, neighbors with the same class label have a lower energy and are *a priori* more likely. An MRF with this interaction energy is also known as a Potts model [47]. Sometimes there is also a distance weight included to account for anisotropic images [46, 60]. However, the standard Potts model does not provide enough flexibility to account for different cases of neighboring tissues. For example, a voxel with gray matter may be neighbor to a voxel with white matter but it cannot be a neighbor to air, yet in the Potts model all neighboring tissues are given the same energetic cost. We can incorporate more flexible prior information with an interaction matrix **J** of size *K* × *K*, whose element *J*_*xx*′_ represents the negative interaction energy between tissue class *x* and *x*′. With this we can define the second order potential *V*_*ij*_ as

$$\begin{array}{c} V_{ij}\left(x_{i},x_{j}\right)=-x_{i}^{T}J_{ij}x_{j}. \end{array}$$ (26)

In conclusion, the two types of priors can be combined into a single joint prior probability

$$\begin{array}{c} P\left(X\right)=\frac{1}{Z}\exp(\frac{1}{2}\sum_{i=1}^{N}\sum_{j\in {𝓝}_{i}}x_{i}^{T}J_{ij}x_{j}+\sum_{i=1}^{N}h_{i}^{T}x_{i}). \end{array}$$ (27)

Eq 27 can be thought of as an extended Potts model. All prior information is now captured by **h**_*i*_ and **J**_*ij*_. In next section we will discuss how these parameters can be set based on truth data from existing segmentations: **h**_*i*_ will be determined by the probability of observing a tissue in a given location, i.e., the “tissue probability map (TPM)”; **J**_*ij*_ will be determined by measuring the co-occurrence of tissues in neigboring locations; we will refer to this as “tissue correlation map (TCM)”.

### B.6 Model parameters set based on prior segmentations

We have argued that the first and second order potentials can be used to define location prior and neighborhood prior. Here we will discuss how one should select the corresponding parameters **h**_*i*_ and **J**_*ij*_ to express this prior knowledge. Prior knowledge is typically available as a set of validated segmentations on historical data. In the context of brain segmentation, with fairly reproducible anatomy across subjects, it is customary to warp individual heads into a single model head, so that specific locations can be identified across different heads. This process is known as “normalization” and is crucial if we want to use prior information on location for segmentation of a given image [66, 67]. A popular standard for such a reference today is MNI-152 head which represents an average over 152 heads compiled by the Montreal Neurological Institute [67, 76]. To explain how these data can serve as prior information to determine **h**_*i*_ and **J**_*ij*_, we turn to the concept of maximum entropy.

Assume we are given the mean and correlation of states **X** across such a “normalized” dataset, i.e., the first and second order moment

$$\begin{array}{c} m_{i}=\left\langle x_{i}\right\rangle , \end{array}$$ (28a)

$$\begin{array}{c} C_{ij}=\left\langle x_{i}x_{j}^{T}\right\rangle , \end{array}$$ (28b)

where ⟨…⟩ refers to the sample average over the observed (validated) segmentations, and *j* ∈ 𝓝_*i*_. Note that this **m**_*i*_ and **C**_*ij*_ is in fact the TPM and TCM mentioned before. To specify this prior information—and nothing else—we are interested in the least informative (maximum entropy) joint probability *P*(**X**) that matches these first and second order moments

$$\sum_{X\in 𝓧}P\left(X\right)x_{i}=m_{i},$$ (29a)

$$\sum_{X\in 𝓧}P\left(X\right)x_{i}x_{j}^{T}=C_{ij}.$$ (29b)

It turns out that this maximum entropy probability distribution has exactly the form of the prior we have selected here as in Eq 27 (following the procedure in [77, 78]). More generally, the maximum entropy distribution that constrains first and second order moments does *not* require any particular Markov property. The parameters **h**_*i*_ and **J**_*ij*_ of the extended Potts model are in fact the Lagrange multipliers to the first and second order constraints for *any* neighborhood arrangement, including a fully connected lattice.

Given **m**_*i*_ and **C**_*ij*_ for all *i*, which we can obtain from prior segmentations, in principle Eq 6 can fully specify the values of **h**_*i*_ and **J**_*ij*_. The problem is that these equations cannot be solved in closed form, except for a few special cases (e.g., [78]). In most instances one has to rely on numerical techniques instead and these are only tractable for small systems consist of up to *N* = 100 sites [79]. At present we have an analytical solution for the inversion from **C**_*ij*_ to **J**_*ij*_ only for the most simple case of two sites, *N* = 2 (see Appendix C)

$$\begin{array}{cc} J_{ij} & =\log[C_{ij}\cdot {\text{diag}}^{-1}\left(m_{j}\right)], \end{array}$$ (30a)

$$\begin{array}{cc} h_{i} & =\frac{1}{2}\log m_{i}. \end{array}$$ (30b)

Here diag^−1^(**m**_*j*_) is the inverse of the diagonal matrix made from vector **m**_*j*_. We have seen that, when interaction between sites is taken into account, the relation between **h**_*i*_ and **m**_*i*_ is not just a logarithm, but scaled by 0.5. We can imagine for an *N*-site lattice, the relation would be even more complicated. Since it is intractable to obtain the analytical solutions for the general *N*-site case, here in this work we take a pragmatic approach by keeping **h**_*i*_ = log **m**_*i*_, but using a free parameter *β* before interaction energy **J**_*ij*_ to balance the relative contribution of the neighborhood prior versus the atlas. Therefore, we get Eq. 5 in the main text, and the prior model Eq 27 has a scalar *β*

$$\begin{array}{c} P\left(X\right)=\frac{1}{Z}\exp(\frac{1}{2}\beta \sum_{i=1}^{N}\sum_{j\in {𝓝}_{i}}x_{i}^{T}J_{ij}x_{j}+\sum_{i=1}^{N}h_{i}^{T}x_{i}). \end{array}$$ (31)

Other parameter settings for the prior model are given by Eqs 6 and 7 in the main text.

### B.7 VEM updating equations: The E-step

With MRI intensity distribution and prior model fully parametrized, we are ready to derive the updating equations of the VEM algorithm. Plugging Eq 20 into Eq 18, it can be shown (see Appendix D) that the free energy function can be written as

$$\begin{array}{cc} F(Q,\theta ) & =\sum_{i=1}^{N}\sum_{x_{i}}q\left(x_{i}\right)[\log q\left(x_{i}\right)-\log P\left(y_{i}|x_{i},\theta \right)\\ & -\frac{1}{2}\beta \sum_{j\in {𝓝}_{i}}\sum_{x_{j}}q\left(x_{j}\right)x_{i}^{T}J_{ij}x_{j}-h_{i}^{T}x_{i}]+\log Z. \end{array}$$ (32)

The E-step requires minimization of Eq 32 with respect to *Q* at fixed ***θ***. As pointed out by [46], the mean-field approximation (Eq 20) suggests an incremental way of implementing this minimization, where the free energy is successively minimized for each voxel *i*, with respect to its corresponding posterior probability *q*(**x**_*i*_) and with all other *q*(**x**_*j*_), *j* ≠ *i* held fixed. Therefore, the estimate of the approximate posterior is updated one voxel at a time with the following optimization

$$\begin{array}{c} q\left(x_{i}\right)\leftarrow \underset{q(x_{i})}{\text{argmin}}\;\;F\left(Q,\theta \right),\text{s.t.}\sum_{x_{i}}q\left(x_{i}\right)=1. \end{array}$$ (33)

In contrast to parallel update, where all *q*(**x**_*i*_) are updated at once, this sequential update is guaranteed to converge [46]. The normalization constraint on this optimization ensures that *q*(**x**_*i*_) remains a valid probability function. The solution to this constrained optimization problem can be obtained using Lagrange multipliers (see Appendix E) as

$$\begin{array}{c} q\left(x_{i}\right)=\frac{P\left(y_{i}|x_{i},\theta \right)\exp[\frac{1}{2}\beta \sum_{j\in {𝓝}_{i}}\sum_{x_{j}}q\left(x_{j}\right)x_{i}^{T}J_{ij}x_{j}+h_{i}^{T}x_{i}]}{\sum_{x_{i}}P\left(y_{i}|x_{i},\theta \right)\exp[\frac{1}{2}\beta \sum_{j\in {𝓝}_{i}}\sum_{x_{j}}q\left(x_{j}\right)x_{i}^{T}J_{ij}x_{j}+h_{i}^{T}x_{i}]}. \end{array}$$ (34)

This is Eq 1 in the main text. It combines three sources of information: 1) the voxel intensities through the conditional (*P*(*y*_*i*_|**x**_*i*_, ***θ***)), 2) the neighborhood prior (**J**_*ij*_) obtained from TCM (Eq 30a), and 3) the anatomical prior (**h**_*i*_) computed from TPM (Eq 24). *β* will be tuned using the data from one subject (Evaluation in the main text). Note that when **J**_*ij*_ = **0**, i.e., no interaction between voxels is assumed, Eq 34 is reduced to Eq 17 by using Eq 24, which is the updating equation only applying the atlas information [14]. On the other hand, when **h**_*i*_ = **0**, i.e., no anatomical prior is used, Eq 34 is the updating equation in most segmentation work involving MRF (but no atlas) to ensure spatial consistency (e.g., [18, 33, 46, 70, 71, 74]).

### B.8 Tissue mixtures

A practical difficulty in MRI brain segmentation is the partial volume effect, i.e., a voxel may contain signals from a number of different tissues. Most segmentation algorithms account for this effect by explicitly modeling the distribution of the voxel intensities containing multiple tissue fractions [29, 33, 73, 80]. In this paper we adopt the approach as in [14], where the partial volume effect is modelled by assuming that a tissue type consists of several similar tissue classes with different MR intensities. For example, the skull can contain cancellous and cortical bone, and the soft tissue may contain fat and muscle. The atlas usually only provides prior information for the tissue type (e.g., skull), but not for those detailed tissue classes (e.g., different layers of bone). For electromagnetic forward modeling, we only aim to label tissue types. We will use variable ${\bar{\text{x}}}_{i}$ to refer to such a tissue *type*, and distinguish this from variable **x**_*i*_, which still refers to individual tissue *class* as before. The model for the intensity for a given tissue type becomes then

$$\begin{array}{c} P\left(y_{i}|{\bar{x}}_{i},\theta \right)=\sum_{x_{i}}P\left(y_{i},x_{i}|{\bar{x}}_{i},\theta \right)=\sum_{x_{i}}\gamma_{x\bar{x}}P\left(y_{i}|x_{i},\theta \right). \end{array}$$ (35)

This can be thought of as Gaussian FM model [18, 45] where the mixing proportion—matrix $\gamma_{x\bar{x}}$—represents a parametrization of the conditional probability $P({\text{x}}_{i}|{\bar{\text{x}}}_{i})=\gamma_{x\bar{x}}$, which is independent of location. This model is quite conventional in segmentation and is also used in SPM8. However, it deviates from the one presented in [14], where the mixing proportion is put before the atlas prior. With this distinction between tissue class labels **X** for which no prior information is available and tissue type labels $\bar{\text{X}}$ for which we have a prior $P(\bar{\text{X}})$ we can redefine the free energy as

$$\begin{array}{c} F\left(Q,\theta \right)=\sum_{X\in 𝓧}\sum_{\bar{X}\in \bar{𝓧}}Q\left(X,\bar{X}\right)\log\frac{Q(X,\bar{X})}{P(X,\bar{X},Y|\theta )}. \end{array}$$ (36)

In the same manner as above this simplifies to

$$\begin{array}{cc} F(Q,\theta ) & =\sum_{i=1}^{N}\sum_{x_{i}}\sum_{{\bar{x}}_{i}}q\left(x_{i},{\bar{x}}_{i}\right)[\log q\left(x_{i},{\bar{x}}_{i}\right)-\log P\left(y_{i},x_{i}|{\bar{x}}_{i},\theta \right)\\ & -\frac{1}{2}\beta \sum_{j\in {𝓝}_{i}}\sum_{x_{j}}\sum_{{\bar{x}}_{j}}q\left(x_{j},{\bar{x}}_{j}\right){\bar{x}}_{i}^{T}J_{ij}{\bar{x}}_{j}-h_{i}^{T}{\bar{x}}_{i}]+\log Z. \end{array}$$ (37)

The estimate of the posterior is now

$$\begin{array}{c} q\left(x_{i},{\bar{x}}_{i}\right)=\frac{\gamma_{x\bar{x}}P\left(y_{i}|x_{i},\theta \right)\exp[\frac{1}{2}\beta \sum_{j\in {𝓝}_{i}}\sum_{{\bar{x}}_{j}}q\left({\bar{x}}_{j}\right){\bar{x}}_{i}^{T}J_{ij}{\bar{x}}_{j}+h_{i}^{T}{\bar{x}}_{i}]}{\sum_{{\bar{x}}_{i}}P\left(y_{i}|{\bar{x}}_{i},\theta \right)\exp[\frac{1}{2}\beta \sum_{j\in {𝓝}_{i}}\sum_{{\bar{x}}_{j}}q\left({\bar{x}}_{j}\right){\bar{x}}_{i}^{T}J_{ij}{\bar{x}}_{j}+h_{i}^{T}{\bar{x}}_{i}]}. \end{array}$$ (38)

Note here Eq 35 is used when deriving the above equation. The desired estimate for the probability of tissue type ${\bar{\text{x}}}_{i}$ is then

$$\begin{array}{c} q\left({\bar{x}}_{i}\right)=\sum_{x_{i}}q\left(x_{i},{\bar{x}}_{i}\right). \end{array}$$ (39)

Eqs 38 and 39 are the Eq 2 in the main text.

### B.9 VEM updating equations: The M-step

The M-step in VEM is to minimize the free energy function Eq 32 (or Eq 37 if using tissue mixtures) with respect to ***θ*** at fixed *Q*.

$$\begin{array}{c} \theta \leftarrow \underset{\theta }{\text{argmin}}\;\;F\left(Q,\theta \right),\text{s.t.}\sum_{x}\gamma_{x\bar{x}}=1. \end{array}$$ (40)

Here ***θ*** is the mean, variance and mixing proportion in the Gaussian mixture, as shown in Eqs 1 and 35. The only constraint is that all the mixing proportions sum to 1 for each tissue type, as dictated by the definition of FM model. Using Lagrange multipliers (see Appendix F), we get

$$\begin{array}{cc} \mu_{x} & =\frac{\sum_{i=1}^{N}q\left(x_{i}=x\right)y_{i}}{\sum_{i=1}^{N}q\left(x_{i}=x\right)}, \end{array}$$ (41a)

$$\begin{array}{cc} \Sigma_{x} & =\frac{\sum_{i=1}^{N}q\left(x_{i}=x\right)\left(y_{i}-\mu_{x}\right){\left(y_{i}-\mu_{x}\right)}^{T}}{\sum_{i=1}^{N}q\left(x_{i}=x\right)}, \end{array}$$ (41b)

$$\begin{array}{cc} \gamma_{x\bar{x}} & =\frac{\sum_{i=1}^{N}q\left(x_{i}=x,{\bar{x}}_{i}=\bar{x}\right)}{\sum_{i=1}^{N}q\left({\bar{x}}_{i}=\bar{x}\right)}. \end{array}$$ (41c)

These are the Eq 1 in the main text. For the sake of generality we have given the Gaussian parameters here for multimodal data. The approximate posteriors $q(x_{i}=x,{\bar{x}}_{i}=\bar{x})$ for specific tissue type $\bar{x}$ and unspecified tissue class *x* are given by Eqs 38 and 39. Note that with update Eq 41c any parameter of matrix $\gamma_{x\bar{x}}$ that is initialized with 0 remains 0 during update. Thus, by initializing matrix $\gamma_{x\bar{x}}$ with only one non-zero entry for each value *x*, each hidden tissue class *x* can only belong to a single specific tissue type $\bar{x}$. This gives the conventional mixture model where the mixture elements only belong to a single mixture.

## C 2-site problem

Consider a trivial 2-site lattice. **x**_1_ and **x**_2_ are the class vectors for the two sites. According to the prior model Eq 27, we can write the distribution of the lattice configuration as

$$\begin{array}{c} P\left(X\right)=\frac{1}{Z}\exp(\frac{1}{2}x_{1}^{T}J_{12}x_{2}+\frac{1}{2}x_{2}^{T}J_{21}x_{1}+h_{1}^{T}x_{1}+h_{2}^{T}x_{2}). \end{array}$$ (42)

To get the expression of **J**_*ij*_ and **h**_*i*_ in terms of **C**_*ij*_ and **m**_*i*_, we need to solve

$$\sum_{X\in 𝓧}[P\left(X\right)x_{i}x_{j}^{T}]=C_{ij},$$ (43a)

$$\sum_{X\in 𝓧}[P\left(X\right)x_{i}]=m_{i}.$$ (43b)

For this 2-site lattice, the sum $\sum_{\text{X}\in 𝓧}$ can be expanded and thus the partition function *Z* can be analytically expressed. When *i* = 1, *j* = 2, plugging the *P*(**X**) in Eq 42 into Eq. 43, we have

$$\begin{array}{cc} \sum_{x_{1}}\sum_{x_{2}}\frac{\exp(\frac{1}{2}x_{1}^{T}J_{12}x_{2}+\frac{1}{2}x_{2}^{T}J_{21}x_{1}+h_{1}^{T}x_{1}+h_{2}^{T}x_{2})x_{1}x_{2}^{T}}{\sum_{x_{1}}\sum_{x_{2}}\exp(\frac{1}{2}x_{1}^{T}J_{12}x_{2}+\frac{1}{2}x_{2}^{T}J_{21}x_{1}+h_{1}^{T}x_{1}+h_{2}^{T}x_{2})} & =C_{12}, \end{array}$$ (44a)

$$\begin{array}{cc} \sum_{x_{1}}\sum_{x_{2}}\frac{\exp(\frac{1}{2}x_{1}^{T}J_{12}x_{2}+\frac{1}{2}x_{2}^{T}J_{21}x_{1}+h_{1}^{T}x_{1}+h_{2}^{T}x_{2})x_{1}}{\sum_{x_{1}}\sum_{x_{2}}\exp(\frac{1}{2}x_{1}^{T}J_{12}x_{2}+\frac{1}{2}x_{2}^{T}J_{21}x_{1}+h_{1}^{T}x_{1}+h_{2}^{T}x_{2})} & =m_{1}. \end{array}$$ (44b)

Note the denominator in Eq. 44 is the expanded partition function *Z*. These two equations can be simplified by using matrix subscripts *k* and *l* (1 ≤ *k* ≤ *K*, 1 ≤ *l* ≤ *K*),

$$\begin{array}{cc} \frac{\exp[\frac{1}{2}{\left(J_{12}\right)}_{kl}+\frac{1}{2}{\left(J_{21}\right)}_{lk}+{\left(h_{1}\right)}_{k}+{\left(h_{2}\right)}_{l}]}{\sum_{k}\sum_{l}\exp[\frac{1}{2}{\left(J_{12}\right)}_{kl}+\frac{1}{2}{\left(J_{21}\right)}_{lk}+{\left(h_{1}\right)}_{k}+{\left(h_{2}\right)}_{l}]} & ={\left(C_{12}\right)}_{kl}, \end{array}$$ (45a)

$$\begin{array}{cc} \frac{\sum_{l}\exp[\frac{1}{2}{\left(J_{12}\right)}_{kl}+\frac{1}{2}{\left(J_{21}\right)}_{lk}+{\left(h_{1}\right)}_{k}+{\left(h_{2}\right)}_{l}]}{\sum_{k}\sum_{l}\exp[\frac{1}{2}{\left(J_{12}\right)}_{kl}+\frac{1}{2}{\left(J_{21}\right)}_{lk}+{\left(h_{1}\right)}_{k}+{\left(h_{2}\right)}_{l}]} & ={\left(m_{1}\right)}_{k}. \end{array}$$ (45b)

By observation, we can immediately get

$$\begin{array}{c} \sum_{l}{\left(C_{12}\right)}_{kl}={\left(m_{1}\right)}_{k}, \end{array}$$ (46)

and similarly,

$$\begin{array}{c} \sum_{k}{\left(C_{12}\right)}_{kl}={\left(m_{2}\right)}_{l}. \end{array}$$ (47)

In order to satisfy Eq 45a, we can guess

$$\begin{array}{c} {\left(h_{1}\right)}_{k}=\frac{1}{2}\log{\left(m_{1}\right)}_{k}, \end{array}$$ (48a)

$$\begin{array}{c} {\left(h_{2}\right)}_{l}=\frac{1}{2}\log{\left(m_{2}\right)}_{l}, \end{array}$$ (48b)

$$\begin{array}{c} {\left(J_{12}\right)}_{kl}=\log\frac{{\left(C_{12}\right)}_{kl}}{\sum_{k}{\left(C_{12}\right)}_{kl}}=\log\frac{{\left(C_{12}\right)}_{kl}}{{\left(m_{2}\right)}_{l}}, \end{array}$$ (48c)

$$\begin{array}{c} {\left(J_{21}\right)}_{lk}=\log\frac{{\left(C_{21}\right)}_{lk}}{\sum_{l}{\left(C_{21}\right)}_{lk}}=\log\frac{{\left(C_{12}\right)}_{kl}}{\sum_{l}{\left(C_{12}\right)}_{kl}}=\log\frac{{\left(C_{12}\right)}_{kl}}{{\left(m_{1}\right)}_{k}}. \end{array}$$ (48d)

Eqs 48c and 48d use the relations in Eqs 46 and 47, and also the fact that (*C*_12_)_*kl*_ = (*C*_21_)_*lk*_. Plugging Eq. 48 into the left hand side of Eq 45a, combining the sum of logarithms into logarithm of the products, and noticing that the terms (*m*_1_)_*k*_, (*m*_2_)_*l*_ are cancelled out, we finally get

$$\begin{array}{c} \frac{{\left(C_{12}\right)}_{kl}}{\sum_{k}\sum_{l}{\left(C_{12}\right)}_{kl}}, \end{array}$$ (49)

which is equal to the right hand side of Eq 45a, because $\sum_{k}\sum_{l}{\left(C_{12}\right)}_{kl}=1$ by definition. Therefore, Eq. 48 satisfies Eq 45a, and thus the solution to the 2-site problem is

$$\begin{array}{c} {\left(J_{ij}\right)}_{kl}=\log\frac{{\left(C_{ij}\right)}_{kl}}{{\left(m_{j}\right)}_{l}}, \end{array}$$ (50a)

$$\begin{array}{c} {\left(h_{i}\right)}_{k}=\frac{1}{2}\log{\left(m_{i}\right)}_{k}. \end{array}$$ (50b)

These give Eqs 30a and 30b.

## D Derivation of the free energy function

Noticing the fact that the probability distribution *Q*(**X**) sums to 1, i.e., $\sum_{\text{X}\in 𝓧}Q(\text{X})=1$, we can write the free energy function *F*(*Q*, ***θ***) in Eq 18 as

$$\begin{array}{c} F\left(Q,\theta \right)=\sum_{X\in 𝓧}Q\left(X\right)\log\frac{Q(X)}{P(X,Y|\theta )}. \end{array}$$ (51)

Expanding the complete data likelihood *P*(**X**, **Y**|***θ***) into the product of the intensity distribution *P*(**Y**|**X**, ***θ***) and the prior *P*(**X**), and plugging Eq 15, Eq 20 and Eq 31 into Eq 51, we get

$$\begin{array}{ccc} F(Q,\theta ) & = & \sum_{X\in 𝓧}\prod_{m}q\left(x_{m}\right)[\sum_{i}\log q\left(x_{i}\right)-\sum_{i}\log P\left(y_{i}|x_{i},\theta \right)\\ & & -\frac{1}{2}\beta \sum_{i}\sum_{j\in {𝓝}_{i}}x_{i}^{T}J_{ij}x_{j}-\sum_{i}h_{i}^{T}x_{i}+\log Z]. \end{array}$$ (52)

The above equation can be simplified, for example,

$$\begin{array}{ccc} & & \sum_{X\in 𝓧}\prod_{m}q\left(x_{m}\right)\sum_{i}\log q\left(x_{i}\right)\\ & & \;=\sum_{x_{1}}\sum_{x_{2}}\cdots \sum_{x_{i}}\cdots \sum_{x_{N}}\prod_{m}q\left(x_{m}\right)\sum_{i}\log q\left(x_{i}\right)\\ & & \;=\sum_{i}\sum_{x_{1}}\sum_{x_{2}}\cdots \sum_{x_{i}}\cdots \sum_{x_{N}}\prod_{m}q\left(x_{m}\right)\log q\left(x_{i}\right)\\ & & \;=\sum_{i}\sum_{x_{1}}\sum_{x_{2}}\cdots \sum_{x_{i-1}}\sum_{x_{i+1}}\cdots \sum_{x_{N}}\prod_{m\neq i}q\left(x_{m}\right)\sum_{x_{i}}q\left(x_{i}\right)\log q\left(x_{i}\right)\\ & & \;=\sum_{i}\prod_{m\neq i}\sum_{x_{m}}q\left(x_{m}\right)\sum_{x_{i}}q\left(x_{i}\right)\log q\left(x_{i}\right) \end{array}$$ (53)

$$\begin{array}{c} \;=\sum_{i}\sum_{x_{i}}q\left(x_{i}\right)\log q\left(x_{i}\right). \end{array}$$ (54)

The fact that $\sum_{{\text{x}}_{m}}q({\text{x}}_{m})=1$ is used from Eq 53 to Eq 54. Similarly, we have

$$\begin{array}{c} -\sum_{X\in 𝓧}\prod_{m}q\left(x_{m}\right)\sum_{i}\log P\left(y_{i}|x_{i},\theta \right)\\ =-\sum_{i}\sum_{x_{i}}q\left(x_{i}\right)\log P\left(y_{i}|x_{i},\theta \right), \end{array}$$ (55a)

$$\begin{array}{ccc} & & \;-\sum_{X\in 𝓧}\prod_{m}q\left(x_{m}\right)\sum_{i}h_{i}^{T}x_{i}=-\sum_{i}\sum_{x_{i}}q\left(x_{i}\right)h_{i}^{T}x_{i},\\ & & \;-\frac{1}{2}\beta \sum_{X\in 𝓧}\prod_{m}q\left(x_{m}\right)\sum_{i}\sum_{j\in {𝓝}_{i}}x_{i}^{T}J_{ij}x_{j} \end{array}$$ (55b)

$$\begin{array}{ccc} & & \;=-\frac{1}{2}\beta \sum_{i}\sum_{x_{i}}q\left(x_{i}\right)\sum_{j\in {𝓝}_{i}}\sum_{x_{j}}q\left(x_{j}\right)x_{i}^{T}J_{ij}x_{j}. \end{array}$$ (55c)

Plugging Eqs 54 and 55 into Eq 52, we get Eq 32.

## E Derivation of the updating equation in the E-step

With the objective function *F*(*Q*, ***θ***) as in Eq 32 and the constraint $\sum_{{\text{x}}_{i}}q({\text{x}}_{i})=1$, we introduce a generalized function using the Lagrange multipliers *λ*_*i*_

$$\begin{array}{c} 𝓕=F\left(Q,\theta \right)+\sum_{i}\lambda_{i}(\sum_{x_{i}}q\left(x_{i}\right)-1). \end{array}$$ (56)

Then we need to minimize 𝓕 with respect to *q*(**x**_*i*_). To avoid notation confusion, we take derivative of 𝓕 with respect to *q*(**z**_*n*_)

$$\begin{array}{ccc} & & \frac{\partial 𝓕}{\partial q(z_{n})}=\frac{\partial F(Q,\theta )}{\partial q(z_{n})}+\sum_{i}\lambda_{i}\sum_{x_{i}}\delta \left(x_{i},z_{n}\right)\\ & & =\sum_{i}\sum_{x_{i}}[\delta \left(x_{i},z_{n}\right)\log q\left(x_{i}\right)+\delta \left(x_{i},z_{n}\right)]\\ & & +\sum_{i}\sum_{x_{i}}\delta \left(x_{i},z_{n}\right)[-\log P(y_{i}|x_{i},\theta )\\ & & -\frac{1}{2}\beta \sum_{j\in {𝓝}_{i}}\sum_{x_{j}}q\left(x_{j}\right)x_{i}^{T}J_{ij}x_{j}-h_{i}^{T}x_{i}]+\lambda_{n}. \end{array}$$ (57)

Note that *q*(**x**_*j*_) is treated as a constant when the above derivative is taken. Simplifying Eq 57 and setting it to 0, we can solve *q*(**z**_*n*_) as

$$\begin{array}{cc} q(z_{n}) & =P\left(y_{n}|z_{n},\theta \right)\exp[\frac{1}{2}\beta \sum_{j\in {𝓝}_{n}}\sum_{x_{j}}q\left(x_{j}\right)z_{n}^{T}J_{nj}x_{j}+h_{n}^{T}z_{n}]\\ & \times \exp(-\lambda_{n}-1). \end{array}$$ (58)

Plugging the above solution into $\sum_{{\text{z}}_{n}}q({\text{z}}_{n})=1$ to enforce normalization, and substituting **z**_*n*_ with **x**_*i*_, we can get the Eq 34.

## F Derivation of the updating equation in the M-step

The M-step is to optimize the free energy function *F*(*Q*, ***θ***) with respect to the parameters ***θ*** subject to the constraint $\sum_{x}\gamma_{x\bar{x}}=1$. For generality, we need to use the one involving tissue mixtures (Eq 37). To enforce the constraint we use again the Lagrange multipliers $\lambda_{\bar{x}}$

$$\begin{array}{c} 𝓕=F\left(Q,\theta \right)+\sum_{\bar{x}}\lambda_{\bar{x}}(\sum_{x}\gamma_{x\bar{x}}-1). \end{array}$$ (59)

For generality, we consider the case of multimodal data, and thus we need to minimize 𝓕 with respect to ***μ***_*x*_, **Σ**_*x*_ and $\gamma_{x\bar{x}}$. To avoid confusion, we take derivative with respect to ***μ***_*z*_

$$\begin{array}{ccc} \frac{\partial 𝓕}{\partial \mu_{z}} & = & -\sum_{i}\sum_{x}\sum_{\bar{x}}q\left(x_{i}=x,{\bar{x}}_{i}=\bar{x}\right)\Sigma_{x}^{-1}(y_{i}-\mu_{x})\delta_{xz}\\ & = & -\sum_{i}\sum_{\bar{x}}q\left(x_{i}=z,{\bar{x}}_{i}=\bar{x}\right)\Sigma_{z}^{-1}(y_{i}-\mu_{z}). \end{array}$$ (60)

Setting this to 0, solving for ***μ***_*z*_, replacing *z* with *x* and using the fact that $q(x_{i}=x)=\sum_{\bar{x}}q(x_{i}=x,{\bar{x}}_{i}=\bar{x})$, we get Eq 41a. Similarly, we can obtain the **Σ**_*x*_ as in Eq 41b.

To get $\gamma_{x\bar{x}}$, we take derivative of Eq 59 with respect to $\gamma_{z\bar{z}}$

$$\begin{array}{cc} \frac{\partial 𝓕}{\partial \gamma_{z\bar{z}}} & =-\sum_{i}\sum_{x}\sum_{\bar{x}}q\left(x_{i}=x,{\bar{x}}_{i}=\bar{x}\right)\frac{\delta_{xz}\delta_{\bar{x}\bar{z}}}{\gamma_{x\bar{x}}}\\ & +\sum_{\bar{x}}\lambda_{\bar{x}}\sum_{x}\delta_{xz}\delta_{\bar{x}\bar{z}}\\ & =-\frac{1}{\gamma_{z\bar{z}}}\sum_{i}q\left(x_{i}=z,{\bar{x}}_{i}=\bar{z}\right)+\lambda_{\bar{z}}. \end{array}$$ (61)

Set this to 0, solve for $\gamma_{z\bar{z}}$ and replace *z* with *x*. Using the normalization condition $\sum_{x}\gamma_{x\bar{x}}=1$, we find $\lambda_{\bar{x}}=\sum_{i}\sum_{x}q(x_{i}=x,{\bar{x}}_{i}=\bar{x})$. Inserting this into $\gamma_{x\bar{x}}$ and using Eq 39, one obtains Eq 41c.
